# Integrating Cooperative Dual Active Sites Onto a Plasmonic Photocatalyst for Synergistic Promotion of the Reduction and Oxidation Half‐Reactions in Ammonia Synthesis

**DOI:** 10.1002/advs.202523036

**Published:** 2026-01-22

**Authors:** Boyuan Wu, Leilei Zhang, Ke An, Penglei Wang, Yini Fang, Baocheng Yang, Jianfang Wang

**Affiliations:** ^1^ Department of Physics The Chinese University of Hong Kong Shatin Hong Kong SAR China; ^2^ Henan Key Laboratory of Nanocomposites and Applications Institute of Nanostructured Functional Materials Huanghe Science and Technology College Zhengzhou Henan China

**Keywords:** activation sites, nitrogen photofixation, plasmon resonance, plasmonic photocatalysis, tungsten oxide

## Abstract

Photocatalytic N_2_ fixation offers a promising sustainable alternative to the energy‐intensive Haber–Bosch process. Since N_2_ fixation involves the reduction and oxidation half‐reactions, understanding their synergistic relationship and achieving their cooperation is crucial for improving the overall efficiency of N_2_ fixation. To fulfill this objective, we have designed a type of Ru‐WO_3−_ *_x_*/CoO*_x_* photocatalyst with respective reduction and oxidation active sites. In this type of photocatalyst, Ru doping not only provides reduction sites to facilitate N_2_ adsorption and activation but also optimizes the band structure by widening the defect band and elevating the Fermi level. Meanwhile, the CoO*_x_* clusters serve as O_2_ evolution sites that can accelerate hole utilization and improve electron–hole separation, thereby enhancing N_2_ reduction. Consequently, the Ru‐WO_3−_ *_x_*/CoO*_x_* photocatalyst gives an impressive NH_3_ production rate of 131.95 µmol g^−1^ h^−1^ in pure water, superior to the photocatalysts with a single type of active site. The optimized catalyst delivers a solar‐to‐chemical conversion efficiency of 0.22% under AM 1.5G light illumination and maintains an apparent quantum efficiency above 0.2% within the entire test range, including the near‐infrared region. This work underscores the mutual influence of the oxidation and reduction half‐reactions in photocatalysis and highlights the cooperation between redox‐active sites.

## Graphical abstract

An efficient N_2_ fixation photocatalyst, Ru‐WO_3−_ *_x_*/CoO*_x_*, is prepared by integrating respective reduction and oxidation sites onto a plasmonic semiconductor. Owing to optimized reaction kinetics and promoted charge carrier separation by the synergistic effect between the two types of active sites, the involved reduction and oxidation half‐reactions are both enhanced, giving a high NH_3_ production rate of 131.95 µmol g^−1^ h^−1^.

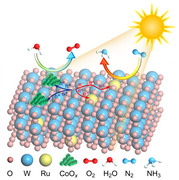

## 1 Introduction

Introduction Ammonia is not only an indispensable feedstock for important agricultural nitrogen fertilizers and industrial chemicals, but also has emerged as a potential hydrogen storage intermediate owing to its high hydrogen capacity and low liquefaction pressure [1, 2]. Currently, the industrial NH_3_ synthesis still predominantly depends on the Haber–Bosch process, which contributes approximately 90% of the global annual NH_3_ production. However, this process requires harsh reaction conditions to activate N_2_, consuming about 1% of the global energy supply and accounting for over 1% of global CO_2_ emissions [3, 4]. Considering the urgent demand for sustainable development, it is highly desirable to develop an environmentally friendly alternative approach for N_2_ fixation into NH_3_. Photocatalytic NH_3_ synthesis has attracted substantial research interest since it can harness inexhaustible solar energy to drive the reaction under mild operating conditions [5, 6, 7]. However, most reported photocatalytic systems exhibit relatively poor NH_3_ yields owing to limited light utilization efficiencies and rapid recombination of photogenerated electron–hole pairs. The rational design of efficient N_2_ fixation photocatalysts with an outstanding carrier generation and utilization capability has remained a daunting challenge [8, 9, 10]

Plasmonic photocatalysts have garnered enormous attention as promising candidates for NH_3_ photosynthesis. Compared with traditional semiconductors, they possess much stronger light absorption owing to localized surface plasmon resonance (LSPR) [11, 12, 13]. Typically, plasmonic catalysts rely on noble metals such as Au and Ag, whose high cost and limited spectral response range severely restrict their practical application prospects. In contrast, degenerately doped semiconductors, such as WO_3−_ *_x_* and MoO_3−_ *_x_*, possess high free carrier densities and strong broadband plasmonic absorption, while being significantly less expensive and more readily synthesized [14]. Moreover, unlike metal–semiconductor hybrids where Schottky barriers at the interface hinder hot carrier transfer, these plasmonic oxides can integrate the hot‐carrier source and catalytic sites within a single phase, allowing excited carriers to be directly transferred to active sites and drive reactions in a Schottky‐barrier‐free manner [15]. As a result, degenerately doped plasmonic semiconductors offer particularly attractive prospects for photocatalytic applications. However, their photocatalytic performances are severely limited by the lack of surface active sites, which leads to sluggish redox kinetics [16]. Although considerable efforts have been dedicated to constructing active sites on plasmonic photocatalysts, most of them focus on N_2_ activation and reduction but overlook the oxidation half‐reaction [17]. However, regulating the water oxidation reaction is equally crucial because the hole‐involved half‐reaction is always quite sluggish and strongly affects charge separation and consumption, resulting in the requirement of hole sacrificial agents in many reported N_2_ fixation systems [18, 19, 20, 21]

Given that the photocatalytic process involves two half‐reactions, the control of reduction–oxidation dual active sites offers a favorable strategy to enhance the overall photocatalytic activity. This approach relies on the integration of distinct but complementary catalytic active sites that allow site‐specific adsorption/activation of reactants and independently promote oxidation and reduction processes [22, 23, 24] By precisely tailoring the chemical and electronic properties of these active sites, synergistic interactions can be achieved to mitigate electron–hole recombination, enhance charge separation, accelerate carrier utilization, and improve the reaction selectivity. Introducing low‐valence active metal species through doping has proven effective for creating active sites for N_2_ reduction [25, 26, 27]. This strategy allows the electronic structure and local coordination environment of materials to be finely controlled to facilitate charge transfer and N_2_ adsorption/activation. Loading water oxidation cocatalysts, such as CoO*_x_* and NiO*_x_*, is a commonly used approach to introduce oxidation active sites to lower the energy barrier for water oxidation and promote electron–hole separation. Although this method is widely employed in photocatalytic overall water splitting to accelerate the reaction kinetics, it has rarely been reported for photocatalytic N_2_ fixation [28, 29, 30]

Herein, we report on the successful integration of both reduction and oxidation active sites onto one photocatalyst through rational and judicious design. Plasmonic WO_3−_ *_x_* nanoplates with Ru doping and surface CoO*_x_* anchoring are constructed as a model material to perform the study. Our investigations reveal that Ru doping not only enhances the LSPR and generates more energetic electrons by expanding the defect band and raising the Fermi level, but also provides efficient reduction sites to facilitate N_2_ chemisorption and N≡N bond activation. Simultaneously, CoO*_x_* loading can significantly promote oxygen evolution, which impels the utilization of photogenerated holes and improves electron–hole separation, thereby accelerating N_2_ reduction. Owing to their synergistic effect, the optimized Ru‐WO_3−_ *_x_*/CoO*_x_* photocatalysts exhibit remarkable NH_3_ and O_2_ evolution rates of 131.95 and 95.7 µmol g^−1^ h^−1^, respectively, under simulated AM 1.5 G light illumination in pure water. This study is distinct from our recent work [17], where a Schottky‐barrier‐free plasmonic photocatalyst was demonstrated with tungsten oxide through hydrogen doping, oxygen‐vacancy introduction, and metal doping. In that work, the kinetics of the oxidation half‐reaction were not specifically investigated by introducing oxidation active sites.

## 2 Results and Discussion

**FIGURE 1:**
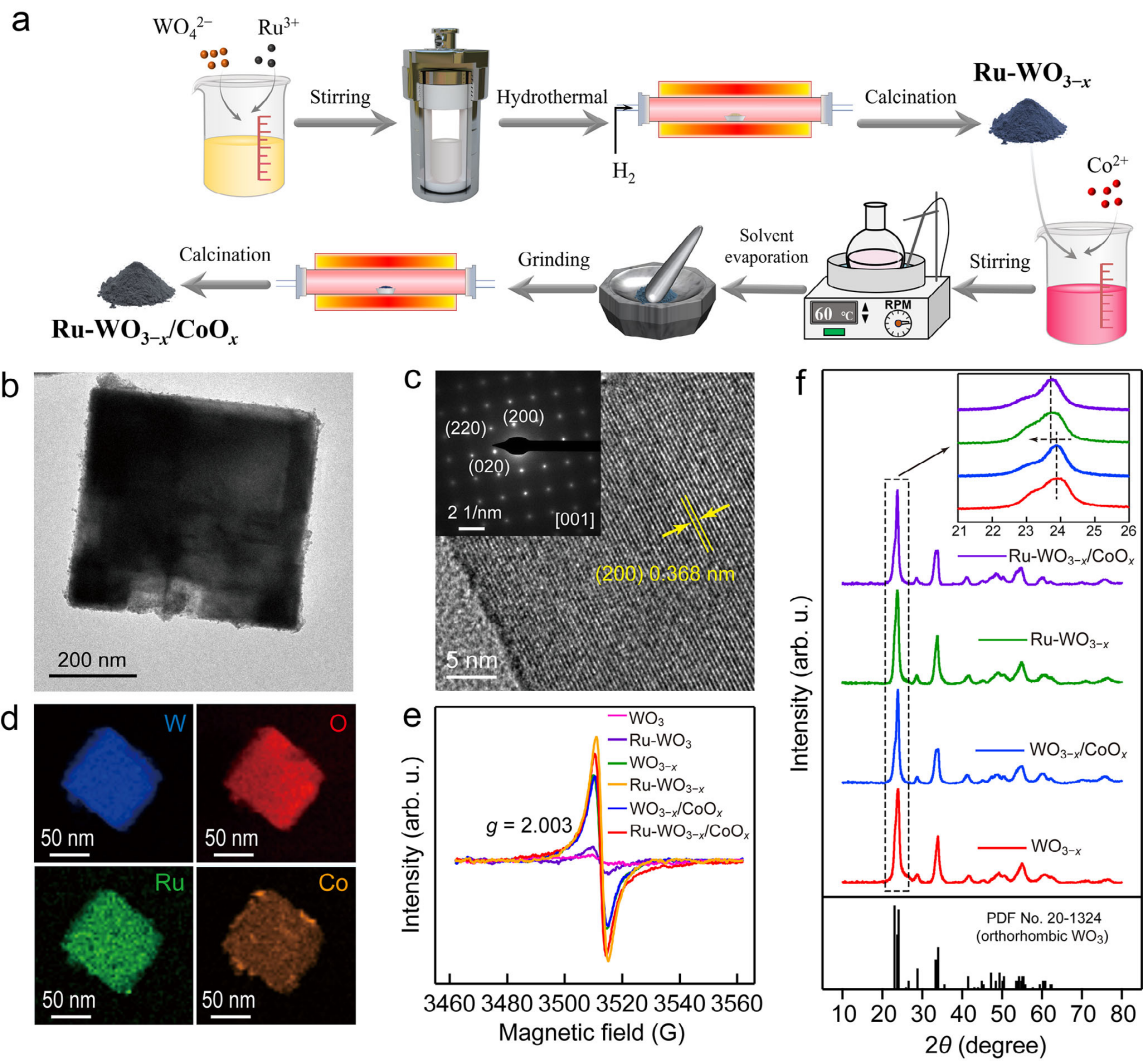
Synthesis route, structure, and morphology characterization. (a) Schematic displaying the synthesis route of Ru‐WO_3−_ *_x_*/CoO*_x_*. (b) TEM image of the Ru‐WO_3−_ *_x_*/CoO*_x_*. (c) HRTEM image and corresponding electron diffraction pattern of Ru‐WO_3−_ *_x_*/CoO*_x_*. (d) EDX mapping images of the different elements in Ru‐WO_3−_ *_x_*/CoO*_x_*. (e, f) EPR spectra (e) and XRD patterns (f) of the WO_3_‐based samples.

The synthesis route of Ru‐WO_3−_ *_x_*/CoO*_x_* is illustrated in Figure 1a. Briefly, Ru‐doped WO_3_ nanoplates (Ru‐WO_3_) were synthesized using a facile hydrothermal method and then annealed in a H_2–_Ar mixture atmosphere to produce Ru‐WO_3−_ *_x_* with abundant oxygen vacancies (OVs). CoO*_x_* loading was achieved by impregnation in a Co^2+^ solution, followed by solvent evaporation and heat treatment. For comparison, WO_3−_ *_x_* and WO_3−_ *_x_*/CoO*_x_* were also synthesized. Scanning electron microscopy (SEM) imaging revealed that the Ru‐doped samples can maintain the square nanoplate morphology of pristine WO_3−_ *_x_* with an average size of ∼500 nm (Figure S1). The rough surfaces and edges in the transmission electron microscopy (TEM) images confirm the loading of ultrasmall CoO*_x_* nanoclusters (Figure 1b and Figure S2). The high‐resolution TEM (HRTEM) images of Ru‐WO_3−_ *_x_*/CoO*_x_* display a clear ordered lattice spacing of 0.368 nm (Figure 1c), which corresponds to the (200) lattice planes of orthorhombic WO_3_ and is consistent with the electron diffraction pattern. Energy‐dispersive X‐ray spectroscopy (EDX) elemental mapping confirmed the uniform distribution of W, O, Ru, and Co in Ru‐WO_3−_ *_x_*/CoO*_x_* (Figure 1d). Their molar fractions in the different samples were determined and summarized (Figure S3 and Table S1). Except for WO_3_ and Ru‐WO_3_, all WO_3_‐based samples exhibit a clear, symmetric resonance peak at *g* = ∼2.003 in the low‐temperature EPR spectra (Figure 1e), which is characteristic of unpaired electrons trapped at OVs and associated low‐valence W‐species in oxygen‐deficient WO_3_ [31]. Compared with the undoped counterparts, the Ru‐doped samples show a significantly enhanced signal intensity, while the *g* value and line shape remain essentially unchanged, indicating that Ru doping increases the OV concentration in the lattice without introducing new paramagnetic species. In addition, the EPR signal intensities of the CoO*_x_*‐loaded samples show no clear difference relative to that of the unloaded samples, indicating that the calcination process in the CoO*_x_* loading process does not lead to substantial re‐oxidation of OVs. This observation aligns with the Raman spectra (Figure S4), where the decrease in the peak intensity indicates the increase in the OV amount. Furthermore, the crystalline structures were investigated by X‐ray diffraction (XRD, Figure 1f). All diffraction peaks can be well indexed to the orthorhombic WO_3_ (JCPDS No. 20–1324) with no peaks attributed to Ru species, suggesting that Ru is doped into the WO_3_ lattice. Notably, the diffraction peaks of the Ru‐doped samples exhibit a shift toward smaller diffraction angles, indicating lattice expansion caused by Ru substitution [32]. X‐ray photoelectron spectroscopy (XPS) results further prove the successful introduction of Ru and CoO*_x_* and confirm the presence of low‐valence W species and oxygen defects (Figure S5 and Table S2–S4).

**FIGURE 2:**
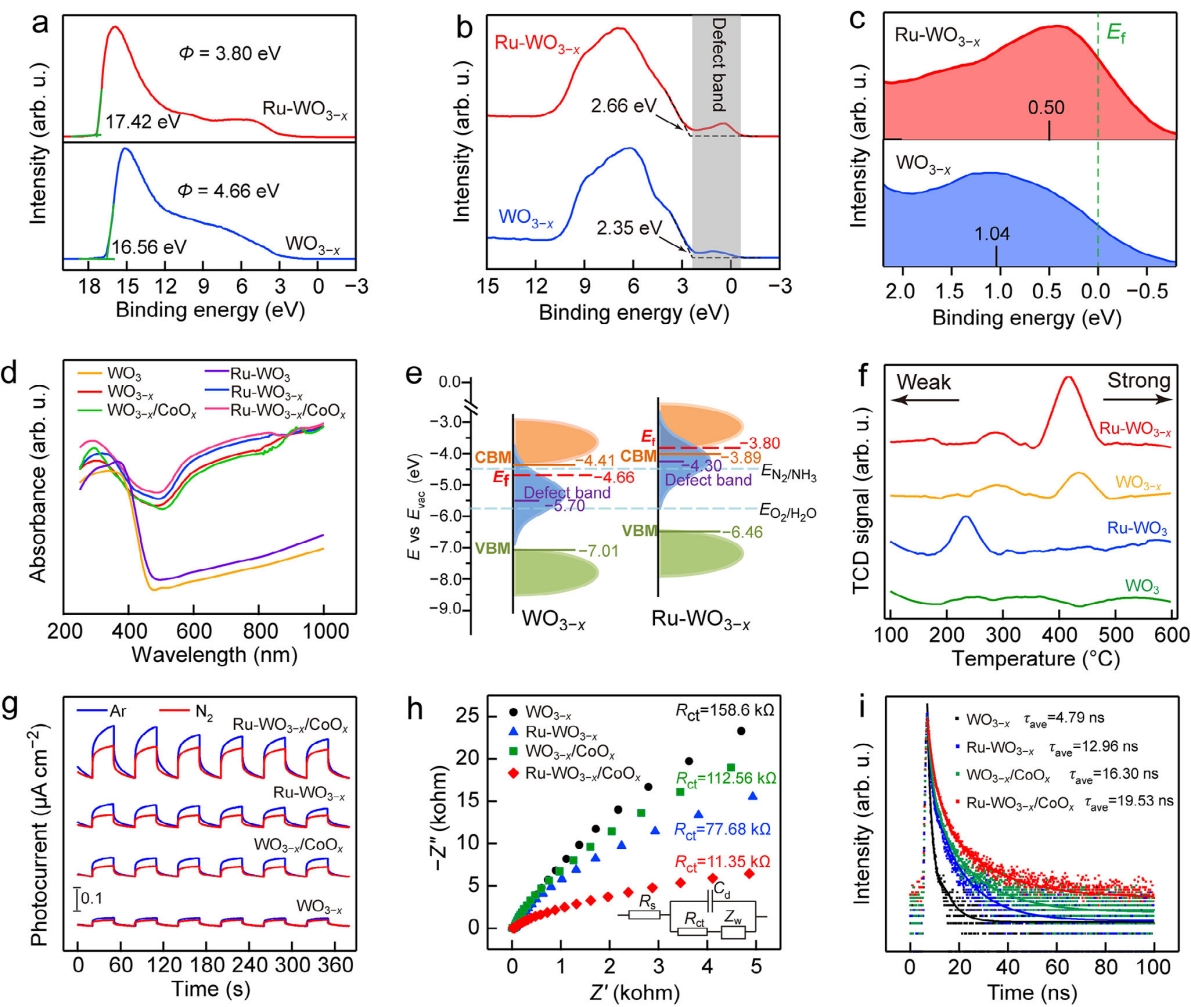
Optical and electronic properties. (a) UPS spectra of WO_3−_ *_x_* and Ru‐WO_3−_ *_x_*. (b, c) VB‐XPS spectra (b) and corresponding defect‐band levels (c) of WO_3−_ *_x_* and Ru‐WO_3−_ *_x_*. (d) UV–Vis–NIR absorption spectra of the WO_3_‐based samples. (e) Schematic illustration of the electronic band structures of WO_3−_ *_x_* and Ru‐WO_3−_ *_x_*. (f) N_2_‐TPD curves of WO_3_, Ru‐WO_3_, WO_3−_ *_x_* and Ru‐WO_3−_ *_x_*. (g–i) Transient photocurrent responses (g), EIS spectra (h), and TRPL spectra (i) of WO_3−_ *_x_*, Ru‐WO_3−_ *_x_*, WO_3−_ *_x_*/CoO*_x_*, and Ru‐WO_3−_ *_x_*/CoO*_x_*.

We further studied the effects of the reductive and oxidative active sites on the optical and electronic properties. The role of Ru doping was first examined systematically. From the ultraviolet photoelectron spectroscopy (UPS) spectra, the work functions (*Φ*) of WO_3−_ *_x_* and Ru‐WO_3−_ *_x_* were found to be 4.66 and 3.80 eV, respectively (Figure 2a). Moreover, the valence band (VB) positions of the samples were determined to be 2.35 and 2.66 eV below the Fermi level (*E*_f_), as shown in the VB‐XPS spectra (Figure 2b). Notably, the sublevels near the Fermi level in the VB‐XPS spectra can be ascribed to the defect band of the degenerate semiconductors, which arises from localized electrons in the *d*‐orbitals of the low‐valence metals with OVs (Figure 2c) [33]. The ultraviolet (UV)–visible (Vis)–near infrared (NIR) absorption spectra show that, compared with the vacancy‐free sample, the oxygen‐deficient samples exhibit a strong and broad absorption tail in the NIR region, which arises from the combined contributions of LSPR and defect‐level‐induced absorption (Figure 2d) [34]. Ru doping further enhances this absorption tail, indicating that Ru incorporation strengthens both the LSPR response and defect formation. The corresponding Tauc plots show that the bandgap of the material decreases upon Ru incorporation (Figure S6) [35]. Based on the above characterization results, the electronic band structures versus the vacuum level (*E*_vac_) were obtained (Figure 2e). Compared to WO_3−_ *_x_*, Ru‐WO_3−_ *_x_* exhibits a similar band structure with a slight upward shift. Notably, Ru doping significantly widens and elevates the defect band, resulting in a higher Fermi level. This allows Ru‐WO_3−_ *_x_* to generate more energetic electrons with a stronger reducing capacity to drive N_2_ reduction [36]. Furthermore, Ru‐WO_3−_ *_x_* possesses a much higher density of free carriers determined by the Mott‐Schottky plot, which favors photocatalytic reactions (Figure S7 and Table S5). Besides the refinement of the electronic structure, the optimization of surface N_2_ adsorption and activation by Ru doping and OVs was examined by temperature‐programmed desorption of N_2_ (N_2_‐TPD), as shown in Figure 2f. The samples without OVs show a desorption peak at ∼250°C, corresponding to the weak physisorption of N_2_ on the surface. In contrast, the samples containing OVs show a strong desorption peak at ∼450°C, indicating the role of surface OVs in N_2_ chemisorption [37]. Notably, the Ru‐doped samples exhibit significantly enhanced desorption peak intensities, suggesting that Ru doping effectively promotes the adsorption of N_2_.

The role of CoO*_x_* in regulating interfacial charge kinetics was also investigated. Ru‐WO_3−_ *_x_*/CoO*_x_* exhibits the highest photocurrent density in both N_2_ and Ar atmospheres (Figure 2g), indicating optimal carrier separation and transport capabilities. The photocurrent response decreases in N_2_ atmosphere, indicating favorable interfacial electron transfer to chemisorbed N_2_ molecules for subsequent N≡N bond activation [38, 39]. The smallest arc in the electrochemical impedance spectra (EIS) suggests the lowest charge transfer resistance of Ru‐WO_3−_ *_x_*/CoO*_x_*, which is consistent with the corresponding fitting results from the equivalent circuit model (Figure 2h). Furthermore, Ru‐WO_3−_ *_x_*/CoO*_x_* displays a much weaker photoluminescence (PL) intensity than the other samples, suggesting its lowest electron–hole recombination rate (Figure S8). Time‐resolved photoluminescence (TRPL) measurements were performed, with the decay curves fitted using a bi‐exponential function (Figure 2i and Table S6) [40]. The results show that both Ru doping and CoO*_x_* loading markedly prolong the carrier lifetime. This behavior can be attributed to the introduction of defect sites by Ru that trap and store photogenerated electrons, as well as the efficient hole extraction by CoO*_x_*, which suppresses electron–hole recombination [41, 42]. Consequently, Ru‐WO_3−_ *_x_*/CoO*_x_* exhibits the longest average lifetime of 19.53 ns, approximately 4.1 times longer than that of pristine WO_3−_ *_x_*, evidencing cooperative electron–hole separation that benefits the photocatalytic reaction.

**FIGURE 3:**
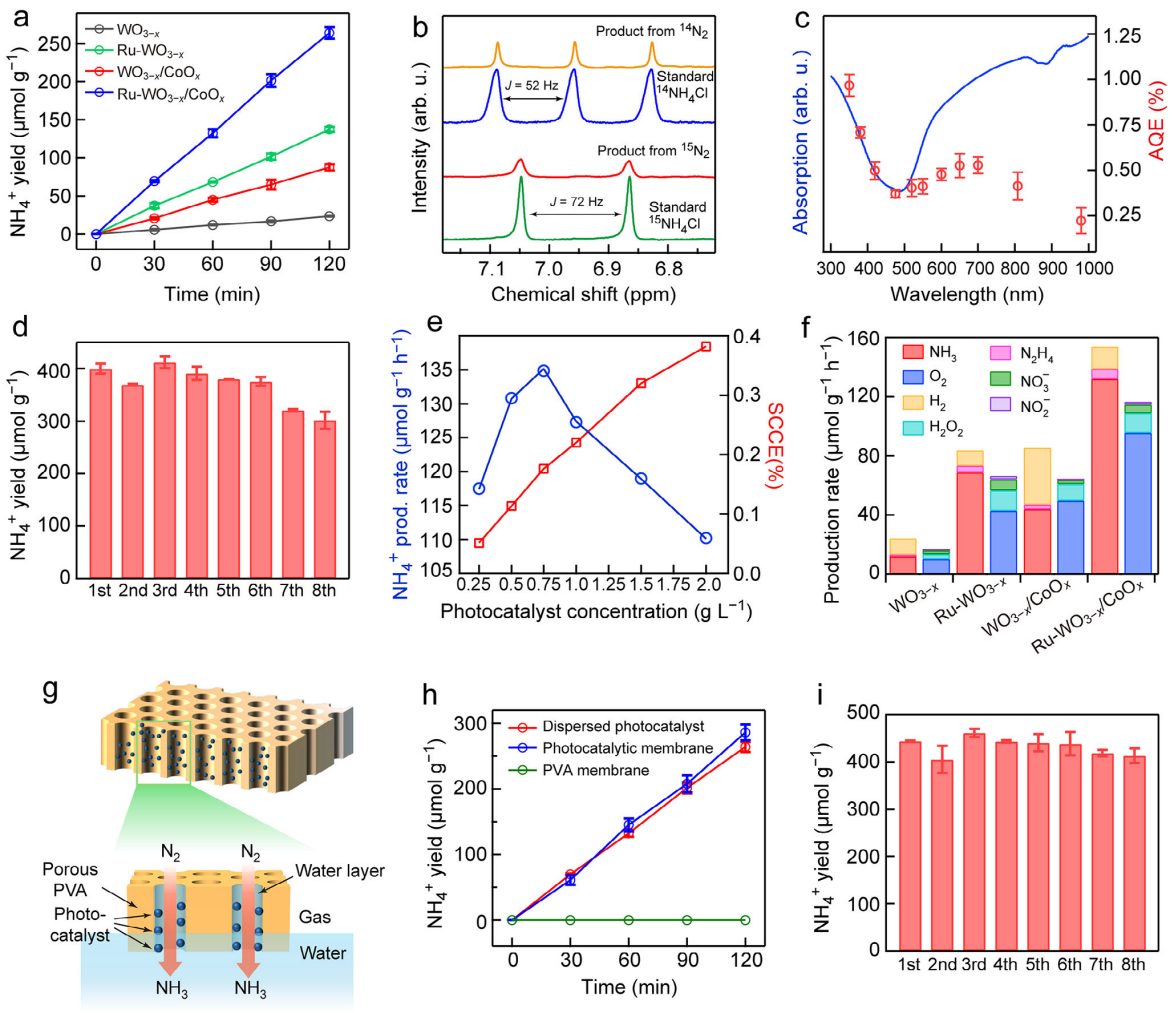
Photocatalysis experiments. (a) Time‐dependent yields of NH_3_ for WO_3−_ *_x_*, Ru‐WO_3−_ *_x_*, WO_3−_ *_x_*/CoO*_x_* and Ru‐WO_3−_ *_x_*/CoO*_x_*. (b) ^1^H NMR spectra of the photocatalytic product using ^14^N_2_ or ^15^N_2_ as the feed gas. (c) Determined AQEs (red dots) for N_2_ fixation over Ru‐WO_3−_ *_x_*/CoO*_x_* under monochromatic light irradiation, in comparison to its light absorption spectrum (blue line). (d) NH_3_ yields of Ru‐WO_3−_ *_x_*/CoO*_x_* in 8 cycles over a continuous 24 h period cycling test. (e) Variations of the SCCE and mass‐normalized NH_3_ production rate with the photocatalyst concentration. (f) Production rates of the different products obtained by WO_3−_ *_x_*, Ru‐WO_3−_ *_x_*, WO_3−_ *_x_*/CoO*_x_*, and Ru‐WO_3−_ *_x_*/CoO*_x_*. (g) Schematic of the prepared photocatalytic membrane. (h) Comparison of the time‐dependent yields of NH_3_ for the prepared photocatalytic membrane and the dispersed powder photocatalyst. (i) NH_3_ yields of the photocatalytic membrane in 8 cycles over a continuous 24 h period cycling test.

The photocatalytic N_2_ fixation performances of the samples were evaluated in N_2_‐saturated pure water under AM 1.5G light illumination (100 mW cm^−2^). Ion chromatography was used to quantify the produced NH_3_ (Figure S9). Time‐dependent measurements revealed that Ru‐WO_3−_ *_x_*/CoO*_x_* exhibits the highest NH_3_ yield after the reaction for 2 h (Figure 3a), giving an average NH_3_ production rate of 131.95 µmol g^−1^ h^−1^, which is about 2 and 3 times higher than those of Ru‐WO_3−_ *_x_* and WO_3−_ *_x_*/CoO*_x_*, respectively (Figure S10). This performance ranks among the best in similar WO_3_‐based photocatalytic systems (Table S7). Moreover, the photocatalytic activity of Ru‐WO_3−_ *_x_*/CoO*_x_* is even highly competitive with previously reported sacrificial‐agent‐free photocatalytic N_2_ reduction systems, which are not limited to WO_3_‐based systems (Table S8). These results unveil that the integration of the reduction and oxidation sites can efficiently promote the photocatalytic NH_3_ synthesis activity of WO_3−_ *_x_*, and a greater promotion can be achieved through their synergistic effect. In addition, the optimal Ru doping and CoO*_x_* loading amounts were determined to be 1 mol% and 3 wt.%, respectively (Figure S11). Comprehensive control experiments were conducted to eliminate the potential interference from external contamination (Figure S12). The results demonstrate that no detectable NH_3_ formation occurred in the absence of any of the following necessary conditions: light, catalyst, nitrogen source (N_2_), or proton source (H_2_O), thereby indicating that NH_3_ was indeed generated from the photocatalytic process. ^15^N_2_ isotope labelling experiments were further conducted to verify the origin of the produced NH_3_. As shown in Figure 3b, the ^1^H nuclear magnetic resonance (NMR) spectrum of the product obtained using ^15^N_2_ feed gas clearly displays a doublet pattern with a coupling constant of *J*_(N─H)_ = 72 Hz, corresponding to standard ^15^NH_4_^+^. In comparison, when ^14^N_2_ was used as the feed gas, a triplet signal with a coupling constant of *J*_(N─H)_ = 52 Hz was observed, which can be assigned to ^14^NH_4_^+^. These results prove that the detected NH_3_ indeed originates from the photocatalytic reduction of N_2_ gas rather than from any nitrogen‐containing contaminants introduced during the reaction. To further verify the reliability of the quantitative results, the NH_3_ yield of Ru‐WO_3−_ *_x_*/CoO*_x_* was also quantified using Nessler's reagent method. The results are in good agreement with those obtained by ion chromatography (Figure S13), providing cross‐validation of the ammonia quantification. We excluded the potential interference from NH_4_^+^ adsorption by conducting NH_4_^+^ adsorption–desorption experiments (Figure S14). No appreciable NH_4_^+^ release was detected after transferring the catalysts, pre‐equilibrated in NH_4_^+^‐containing solutions, into pure water. This result indicates that NH_4_^+^ adsorption on the catalysts is negligible and does not interfere with NH_3_ quantification.

The wavelength‐dependent apparent quantum efficiencies (AQEs) of Ru‐WO_3−_ *_x_*/CoO*_x_* correlate well with its absorption spectrum in the UV–vis range, with a maximal value of 0.96% obtained at 350 nm (Figure 3c). Notably, the catalyst exhibits a comparable AQE at 808 nm to that observed in the visible range. Even at 980 nm, where strong water absorption may diminish the effective photon flux reaching the catalyst surface, the AQE remains as high as 0.22%. These results demonstrate that plasmon‐excited electrons can efficiently drive NH_3_ formation in the NIR region, providing direct evidence that LSPR makes a substantial contribution to the overall photocatalytic activity. Owing to the strong absorption in the visible and NIR regions, Ru‐WO_3−_ *_x_*/CoO*_x_* can deliver a great NH_3_ production activity under UV‐filtered irradiation (*λ* > 420 nm), as shown in Figure S15. A 24 h cycling test (8 cycles) was carried out to evaluate the stability of the Ru‐WO_3−_ *_x_*/CoO*_x_* catalyst (Figure 3d). The photocatalytic activity remained relatively stable during the first 18 h and then exhibited a slight decline. Nevertheless, the NH_3_ production rate was still maintained above 100 µmol g^−1^ h^−1^ throughout the test, highlighting its good reusability and durability. The TEM images and XRD patterns show no pronounced differences before and after the reaction, indicating that its morphology and crystal structure remained unchanged (Figure S16a,b). The XPS peak positions and line shapes of Ru and Co were almost identical to those before the reaction, suggesting that their electronic states and coordination environments were well preserved without noticeable oxidation/reduction (Figure S16c,d). In addition, the ICP‐OES analysis results reveal minor leaching of Ru and Co species during the prolonged reaction (Table S9), which could contribute to the slight activity decay. However, overall, the stability of this catalyst is superior to that of most reported catalysts. The solar‐to‐chemical conversion efficiencies (SCCEs) were determined at different sample concentrations (Figure 3e). At a concentration of 1 g L^−1^, the photocatalytic system gives both a relatively high mass‐normalized activity and an SCCE of 0.22%, making it promising for practical production.

In addition to the target product NH_3_, we also quantitatively analyzed other possible oxidation products (O_2_, H_2_O_2_, NO_3_^−^, NO_2_^−^) and reduction products (H_2_, N_2_H_4_) formed during the reaction (Figure 3f and Table S10). The nitrogen selectivity, defined as the percentage of nitrogen atoms contained in the produced NH_3_ relative to the total number of nitrogen atoms in all detected nitrogen‐containing products, was also calculated for all catalysts. In the absence of Ru sites, WO_3−_ *_x_* and WO_3−_ *_x_*/CoO*_x_* are unable to effectively utilize photogenerated electrons for N_2_ reduction, and a substantial fraction of electrons is instead consumed in H_2_ evolution. This finding highlights the critical role of Ru sites in N_2_ adsorption and activation. For all samples, O_2_ is identified as the dominant oxidation product (Figure S17). CoO*_x_* loading significantly enhances the O_2_ evolution rate and improves the hole‐utilization efficiency, which in turn promotes the reduction half‐reaction. At the same time, it enables the selective consumption of holes for water oxidation, thereby suppressing the competitive oxidation of N_2_ and improving the nitrogen selectivity. Owing to the cooperative effect of the two types of active sites, Ru‐WO_3−_ *_x_*/CoO*_x_* exhibits the highest yields for both NH_3_ and O_2_, as well as the highest nitrogen selectivity among all catalysts. Furthermore, electron‐balance analysis confirms the kinetic matching between the oxidation and reduction half‐reactions, indicating that the CoO*_x_*‐induced enhancement arises from a genuine improvement of the oxidation half‐reaction rather than from an uncontrolled hole‐scavenging process. Control experiments using sacrificial agents provided additional insights. The enhancement in NH_3_ production achieved by loading CoO*_x_* onto Ru‐WO_3__−__x_ is comparable to that obtained by adding methanol as a hole scavenger (Figure S18a), demonstrating that oxidation active sites can effectively replace sacrificial agents to enable efficient N_2_ fixation. Moreover, Ru doping also promotes O_2_ evolution, similar to the effect of adding AgNO_3_ as an electron scavenger (Figure S18b). Taken together, these results prove the mutual enhancement between the oxidative and reductive half‐reactions, highlighting the importance of the dual active site strategy.

It has been reported that O_2_, the targeted oxidation product in our system, may, in principle, compete with N_2_ for electrons and/or adsorption sites to lower the NH_3_ productivity in some photocatalytic N_2_ fixation systems [43, 44]. In Ru‐WO_3−_ *_x_*/CoO*_x_*, however, the reduction and oxidation half‐reactions proceed on spatially distinct sites, where N_2_ adsorption and reduction occur at the Ru sites, whereas O_2_ evolution takes place on the CoO*_x_* nanoclusters. The spatial separation of the reduction and oxidation centers effectively suppresses this competition. Accordingly, Ru‐WO_3−_ *_x_*/CoO*_x_* shows the highest NH_3_ and O_2_ evolution rates and an almost linear NH_3_ yield‐time profile (Figure 3a), indicating that the benefits of accelerated hole consumption and improved charge separation outweigh any inhibitory effect of in situ generated O_2_. In addition, control experiments in air did not show a significant decrease in the NH_3_ production rate (Figure S19), confirming that even in the presence of excess O_2_, the competing O_2_ reduction has only a minor impact and the catalyst still maintains high electron selectivity toward NH_3_ formation.

The recovery and recycling challenges of photocatalysts in the suspended particulate system limit their practical applications. To address this, a floatable catalyst membrane was prepared to highlight its potential for practical use by loading the Ru‐WO_3−_ *_x_*/CoO*_x_* photocatalyst particles onto polyvinyl alcohol (PVA) film through a method reported previously by us [45]. The PVA film possesses a porous structure characterized by a network of interconnected channels (Figure S20a, b), which can accommodate catalyst particles and allow reactants to penetrate deep into the film and access the catalyst particles, enabling sufficient contact and effective mass transport (Figure S20c, d). When the membrane is floated on water, a thin water layer is adsorbed across the film to establish a gas–liquid biphasic system for the N_2_ fixation reaction on the photocatalyst surfaces (Figure 3g). The floatable film can be easily removed from the reaction medium and reused without noticeable catalyst loss. This configuration not only overcomes the challenge of recovering powder catalysts but also improves the photocatalytic stability [46]. As shown in Figure 3h, the NH_3_ yield of the photocatalytic membrane (142.89 µmol g^−1^ h^−1^) is slightly higher than that of the dispersed Ru‐WO_3−_ *_x_*/CoO*_x_* powder. We attribute the comparable mass‐normalized performances of the membrane catalyst and the powder catalyst to two competing factors. On the one hand, the floating membrane shortens the diffusion path of gas molecules, enlarges the effective mass‐transfer area at the gas–liquid–solid interface, and increases the local N_2_ concentration near the catalytic sites. On the other hand, during the membrane synthesis process, it is inevitable that some catalyst particles become deeply embedded within the internal skeleton of the polymer framework rather than being dispersed in the pores, which may reduce the proportion of catalytically accessible sites. Even so, we still consider the photocatalytic membrane developed here to be of considerable significance, as it demonstrates the feasibility of integrating a powder catalyst into a recyclable, scalable module without compromising its intrinsic activity.

To evaluate the long‐term durability of the floating Ru‐WO_3−_ *_x_*/CoO*_x_* photocatalytic membrane, we further performed a continuous 24 h (8 cycles) photocatalytic N_2_ fixation experiment. As shown in Figure 3i, the NH_3_ production rate remained essentially stable over 24 h, with the average NH_3_ production rate within the final cycle comparable to that obtained in the initial cycle. The post‐reaction SEM image and photograph (Figure S21) reveal that the membrane morphology and the porous structure were well preserved after 24 h, with no clear fracture and dissolution. The comparison between Figure 3d, i reveals that the membrane catalyst exhibits superior stability relative to the powder catalyst, indicating that immobilizing the catalyst within a floatable polymer matrix is more beneficial for maintaining its stability than directly dispersing it in an aqueous solution, highlighting the robustness of PVA‐supported Ru‐WO_3−_ *_x_*/CoO*_x_* for practical photochemical systems.

**FIGURE 4:**
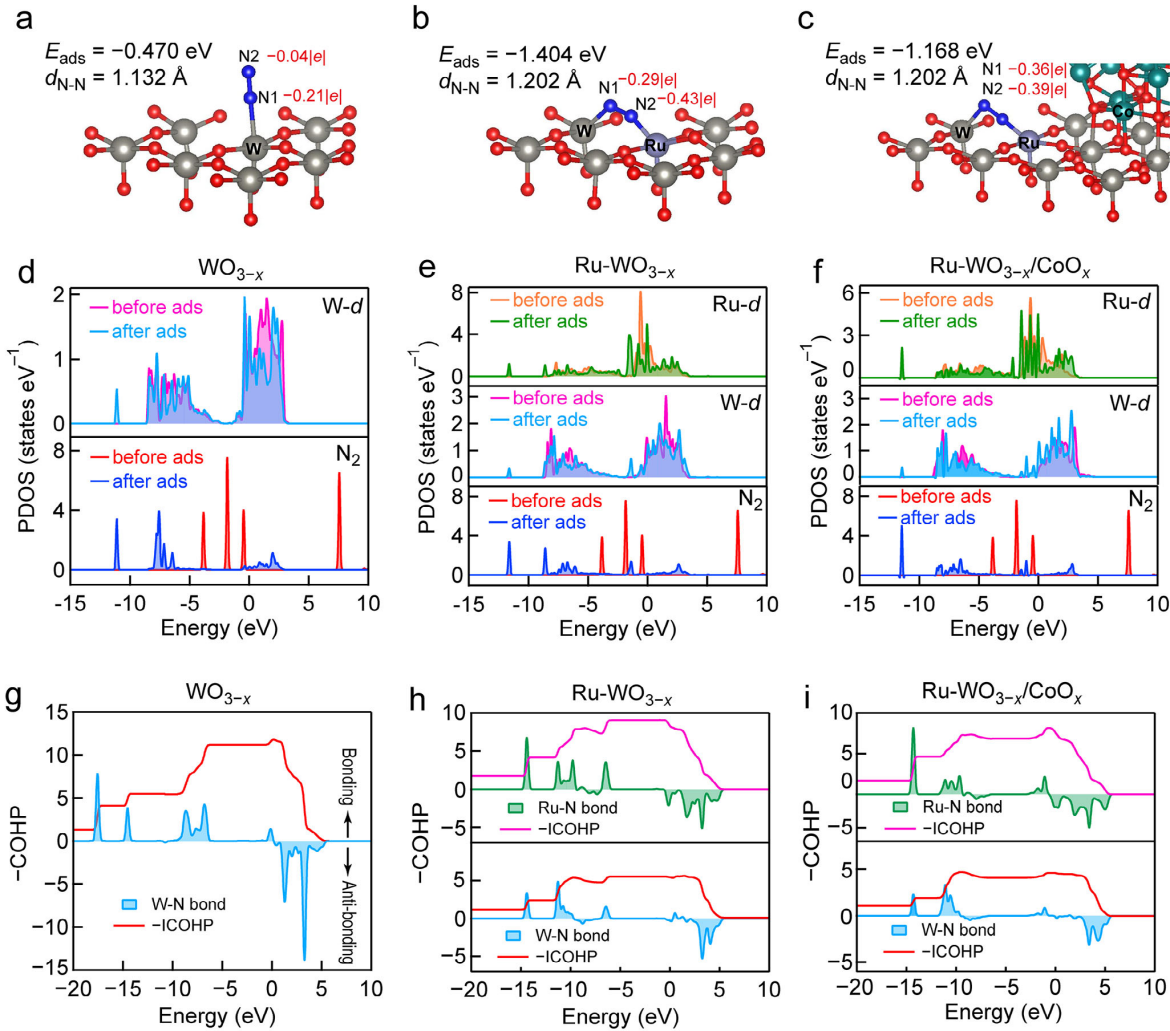
DFT calculations. (a–c) Optimized configurations for N_2_ adsorption on WO_3−_ *_x_* (a), Ru‐WO_3−_ *_x_* (b), and Ru‐WO_3−_ *_x_*/CoO*_x_* (c). (d–f) Comparison of PDOS for WO_3−_ *_x_* (d), Ru‐WO_3−_ *_x_* (e), and Ru‐WO_3−_ *_x_*/CoO*_x_* (f) before and after N_2_ adsorption. (g–i) COHP and ICOHP for the metal–N bond in WO_3−_ *_x_* (g), Ru‐WO_3−_ *_x_* (h), and Ru‐WO_3−_ *_x_*/CoO*_x_* (i).

Density functional theory (DFT) calculations were further performed to uncover the intrinsic mechanism of N_2_ reduction. Because the adsorption of N_2_ is a precondition for NH_3_ synthesis, the optimized configurations for N_2_ adsorption on the different samples were calculated (Figure S22). Compared to pristine WO_3−_ *_x_* (Figure 4a), Ru‐WO_3−_ *_x_* can effectively adsorb N_2_ in two modes, in which the side‐on mode in the Ru–N–N–W configuration is more favorable with an adsorption energy (*E*_ads_) of −1.404 eV, proving the critical role of Ru sites in enhancing N_2_ adsorption (Figure 4b). Further calculations verified that the side‐on configuration can also be achieved when the CoO*_x_* nanocluster is near the adsorption site (Figure 4c). The N–N bond length on the pristine WO_3−_ *_x_* remains nearly unchanged compared to the N≡N bond (1.10 Å) in free N_2_. In contrast, the N–N bond on Ru‐WO_3−_ *_x_* is significantly elongated to 1.202 Å, revealing the successful activation of N_2_ molecules. Bader charge analysis revealed that the total electron transfer from WO_3−_ *_x_* to N_2_ after adsorption is only 0.2*e*, while this value exceeds 0.7*e* for both Ru‐WO_3−_ *_x_* and Ru‐WO_3−_ *_x_*/CoO*_x_*, indicating more efficient electron transfer between the metal atoms and N_2_ on these samples.

Projected density of states (PDOS) analysis further supported these findings. In comparison to WO_3−_ *_x_*, Ru‐WO_3−_ *_x_* and Ru‐WO_3−_ *_x_*/CoO*_x_* exhibit significant changes in PDOS, with new states appearing near the Fermi level during N_2_ adsorption, indicating stronger metal–N orbital hybridization (Figure 4d–f). In particular, the PDOS changes in the *d* orbitals of Ru are more remarkable than those in the *d* orbitals of W, suggesting a stronger interaction between Ru and N. From the perspective of the N_2_ molecule, adsorption on Ru‐WO_3−_ *_x_* and Ru‐WO_3−_ *_x_*/CoO*_x_* reduces the unoccupied anti‐bonding states above the Fermi level, leading to more bonding electrons and a higher metal–N bond order. Crystal orbital Hamilton population (COHP) was calculated to evaluate the strength of metal–N bonds (Figure 4g–i). In all samples, the metal–N bonds present negative values of the integrated COHP (ICOHP), with substantial bonding states below the Fermi level, confirming effective bonding between metal and nitrogen. All of the computational results provide strong theoretical evidence for the significant role of Ru doping in N_2_ adsorption and activation. Moreover, the similar results of Ru‐WO_3−_ *_x_* and Ru‐WO_3−_ *_x_*/CoO*_x_* demonstrate that CoO*_x_* facilitates N_2_ reduction by accelerating the oxidation reaction instead of directly influencing the thermodynamics and kinetics of N_2_ reduction.

## 3 Conclusion

In summary, we have successfully integrated reduction active sites (Ru) and oxidation active sites (CoO*_x_*) onto plasmonic WO_3−_ *_x_* nanoplates to construct an efficient photocatalyst for N_2_ fixation to NH_3_. Comprehensive experiments and theoretical calculations have revealed that the synergistic effect between the two types of active sites significantly promotes the N_2_ fixation performance. Specifically, Ru doping not only widens the defect band and elevates the Fermi level to provide energetic electrons, but also provides active sites for N_2_ chemisorption and N≡N bond activation. At the same time, CoO*_x_* nanoclusters act as an O_2_ evolution cocatalyst to accelerate the oxidation kinetics and promote electron–hole separation and utilization. Therefore, Ru‐WO_3−_ *_x_*/CoO*_x_* gives a high NH_3_ production rate of 131.95 µmol g^−1^ h^−1^ and selectivity over 90%. Our work highlights an effective strategy to optimize photocatalytic performances and demonstrates the importance of regulating the oxidation half‐reaction in N_2_ fixation.

## Conflicts of Interest

The authors declare no conflict of interest.

## Supporting information




**Supporting File**: advs74026‐sup‐0001‐SuppMat.docx.

## Data Availability

The data that support the findings of this study are available from the corresponding author upon reasonable request.
