# Supplementary material for: Integrating Cooperative Dual Active Sites Onto a Plasmonic Photocatalyst for Synergistic Promotion of the Reduction and Oxidation Half‐Reactions in Ammonia Synthesis
Source: Adv Sci (Weinh). 2026 Jan 22;13(19):e23036. doi: 10.1002/advs.202523036 (PMC13045322; doi:10.1002/advs.202523036)
Supplement: Supplementary file 1 — Supporting File: advs74026‐sup‐0001‐SuppMat.docx. [file ADVS-13-e23036-s001.docx]

**Integrating Cooperative Dual Active Sites onto a Plasmonic Photocatalyst for Synergistic Promotion of the Reduction and Oxidation Half-Reactions in Ammonia Synthesis**

Boyuan Wu, Leilei Zhang, Ke An, Penglei Wang, Yini Fang, Baocheng Yang,* and Jianfang Wang*

*Correspondence author. E-mail: jfwang@phy.cuhk.edu.hk (J. F. Wang); baochengyang@infm.hhstu.edu.cn (B. C. Yang).

**Supporting Information**

**Table of Contents**

**1. Experimental Section**

**1.1. Materials and Characterization**

**1.2. Synthesis of WO_3−_*_x_* and Ru-WO_3−_*_x_***

**1.3. Synthesis of WO_3−_*_x_*/CoO*_x_* and Ru-WO_3−_*_x_*/CoO*_x_***

**1.4. Preparation of Ru-WO_3−_*_x_*/CoO*_x_***-**Loaded PVA Membranes**

**1.5. Photocatalytic N_2_ Fixation Tests**

**1.6. AQE and SCCE Determination**

**1.7. Photoelectrochemical Experiments**

**1.8. ^15^N_2_ Isotope Labelling Experiments**

**1.9. NH_4_^+^ Adsorption–Desorption Experiment**

**1.10. DFT Calculations**

**1.11. Statistical Analysis**

1. **Supplementary Figures**

**Figure S1.** SEM images of WO_3−_*_x_*, Ru-WO_3−_*_x_*, WO_3−_*_x_*/CoO*_x_*, and Ru-WO_3−_*_x_*/CoO*_x_*.

**Figure S2.** TEM images of WO_3−_*_x_*, Ru-WO_3−_*_x_*, and WO_3−_*_x_*/CoO*_x_*.

**Figure S3.** EDX spectra of WO_3−_*_x_*, Ru-WO_3−_*_x_*, WO_3−_*_x_*/CoO*_x_*, and Ru-WO_3−_*_x_*/CoO*_x_*.

**Figure S4.** Raman spectra of the WO_3_-based samples.

**Figure S5.** XPS spectra of W 4*f*, O 1*s*, Ru 3*d*, and Co 2*p*.

**Figure S6.** Tauc plots for WO_3_, Ru-WO_3_, WO_3−_*_x_*, and Ru-WO_3−_*_x_*.

**Figure S7.** Mott–Schottky plots of WO_3_, Ru-WO_3_, WO_3−_*_x_*, and Ru-WO_3−_*_x_*.

**Figure S8.** PL spectra of WO_3_, Ru-WO_3_, WO_3−_*_x_*, and Ru-WO_3−_*_x_*.

**Figure S9.** Standard calibration curve for determining ammonia concentrations by ion chromatography.

**Figure S10.** NH_3_ production rates of WO_3−_*_x_*, Ru-WO_3−_*_x_*, WO_3−_*_x_*/CoO*_x_*, and Ru-WO_3−_*_x_*/CoO*_x_*.

**Figure S11.** Optimization of the Ru doping amount and CoO*_x_* loading amount.

**Figure S12.** NH_3_ production rates of Ru-WO_3−_*_x_*/CoO*_x_* under different control experimental conditions.

**Figure S13.** Comparison of the NH_3_ production rates of Ru-WO_3−_*_x_*/CoO*_x_* determined by ion chromatography and Nessler’s reagent.

**Figure S14.** NH_4_^+^ desorption rate of pre-equilibrated WO_3−_*_x_*, Ru-WO_3−_*_x_*, WO_3−_*_x_*/CoO*_x_*, and Ru-WO_3−_*_x_*/CoO*_x_* in pure water.

**Figure S15.** Time-dependent NH_3_ yields of Ru-WO_3−_*_x_*/CoO*_x_* under different light illumination.

**Figure S16.** Stability evaluation of Ru-WO_3−_*_x_*/CoO*_x_*.

**Figure S17.** O_2_ evolution performance.

**Figure S18.** Control experiments by adding sacrificial agents.

**Figure S19.** Comparison of the time-dependent NH_3_ yields of Ru-WO_3−_*_x_*/CoO*_x_* under N_2_ and air atmosphere.

**Figure S20.** Characterization of the photocatalytic membrane.

**Figure S21.** Characterization of the photocatalytic membrane after cycling experiment.

**Figure S22.** Structure models for the DFT calculations.

1. **Supplementary Tables**

**Table S1.** EDX analysis results of element compositions in the different samples.

**Table S2.** Fitting parameters of W 4*f* XPS spectra.

**Table S3.** Fitting parameters of Ru 3*p* XPS spectra.

**Table S4.** Fitting parameters of Co 2*p* XPS spectra.

**Table S5.** Estimated charge carrier densities of the different samples.

**Table S6.** Fitting results for the TRPL spectra of different photocatalysts.

**Table S7.** Photocatalytic nitrogen fixation performances of reported WO_3_-based materials.

**Table S8.** Performance of other reported sacrificial agent-free photocatalytic nitrogen fixation system.

**Table S9.** ICP-OES analysis results of Ru-WO_3−_*_x_*/CoO*_x_* before and after the cycling experiments.

**Table S10**. Summary of production rates, nitrogen selectivity, and electron gain/loss conservation analysis for different catalysts.

1. **References**
2. **Experimental Section**

**1.1. Materials and Characterization**

Sodium tungstate dihydrate (Na_2_WO_4_•2H_2_O, 99%), ruthenium (III) chloride hydrate (RuCl_3_•*x*H_2_O, 35.0%–42.0% Ru basis), tetrafluoroboric acid (HBF_4_, 40 wt% in water), cobalt disodium ethylenediaminetetraacetate (Co-EDTA-Na_2_, 98%) were purchased from Shanghai Aladdin Bio-Chem Technology Co. Ltd. Polyvinyl alcohol (PVA), polyethylene glycol (PEG) and sodium sulfate (Na_2_SO_4_, 99.9%) were purchased from Sigma-Aldrich. Deionized water (Millipore Milli-Q grade, 18.2 MΩ) was used in all experiments. All of the chemical reagents were used as received without further purification.

The morphologies of the synthesized products were investigated by field-emission scanning electron microscopy (SEM, JSM-7800F, JEOL), transmission electron microscopy (TEM, Tecnai-12, FEI), and high-resolution transmission electron microscopy (HRTEM, Tecnai G2 TF20, FEI). Powder X-ray diffraction (XRD) measurements were carried on a diffractometer (SmartLab, Rigaku) equipped with Cu Kα radiation at 40 kV and 80 mA. Surface chemical states and valence band spectra were analyzed by X-ray photoelectron spectroscopy (XPS) on an XPS spectrometer (ESCALAB 250, Thermo Scientific). The work functions of the materials were obtained through ultraviolet photoelectron spectroscopy (UPS) on a Thermo Scientific ESCALAB XI+ spectrometer. An ultraviolet–visible spectrophotometer (Lambda 950, PerkinElmer) equipped with an integrating sphere was employed to record the diffuse reflectance spectra with BaSO_4_ as a reference. A fluorescence spectrometer (FLS1000, Edinburgh) was used for photoluminescence (PL) tests. Time-resolved photoluminescence (TRPL) spectra were recorded on an Edinburgh LifeSpec II fluorescence spectrophotometer equipped with 375 nm pulsed laser. Raman spectra were measured on a Raman spectrometer (inVia Qontor Micro, Renishaw). Low-temperature electron paramagnetic resonance (EPR) spectra were recorded on an EPR spectrometer (EMXnano, Bruker). Nitrogen temperature-programmed desorption (N_2_-TPD) measurements were carried out on a multifunction chemisorption analyzer (AutoChemII2920, Micromeritics) with a ramping rate of 10 °C min^−1^. ^1^H nuclear magnetic resonance (NMR) spectra were taken on a NMR spectrometer (Bruker Avance NEO 600 MHz).

**1.2. Synthesis of WO_3−_*_x_* and Ru-WO_3−_*_x_***

A solution was prepared by dissolving Na_2_WO_4_•2H_2_O (3.6 mmol) in deionized water (60 mL) in advance. Subsequently, HBF_4_ (40 wt%, 3.5 mL) was rapidly injected into the solution with continuous magnetic stirring. After being stirred for 10 min, the yellow mixture was transferred into a 100 mL Teflon-lined stainless steel autoclave and kept in an oven at 160 °C for 12 h. After being cooled down to room temperature, the resultant product was collected by centrifugation and washed with deionized water three times, followed by drying at 60 °C overnight to obtain WO_3_ nanoplates. To synthesize Ru-doped WO_3_, RuCl_3_•*x*H_2_O solution (1 g L^−1^) was used as the Ru precursor and added to the mixture after the addition of HBF_4_. The molar percentage of Ru relative to the total molar amount of W and Ru was set at 0.5%, 1%, 2%, and 3%, respectively.

Heat treatment was employed to introduce oxygen vacancies (OVs). The synthesized WO_3_ and Ru-WO_3_ were annealed at 400 °C for 2 h at a ramping rate of 5 °C min^−1^ in H_2_/Ar (10 vol%/90 vol%) atmosphere to obtain WO_3−_*_x_* and Ru-WO_3−_*_x_*.

**1.3 Synthesis of WO_3−_*_x_*/CoO*_x_* and Ru-WO_3−_*_x_*/CoO*_x_***

50 mg of WO_3−_*_x_* was dispersed in a certain amount of Co-EDTA-Na_2_ aqueous solution (0.75 mg L^−1^) by ultrasonication and stirred for 3 h. The solvent was subsequently evaporated by rotary evaporation at 60 °C. The resultant powder was then annealed at 200 °C for 2 h at a ramping rate of 5 °C min^−1^ in a muffle furnace to obtain WO_3−_*_x_*/CoO*_x_*. Ru-WO_3−_*_x_*/CoO*_x_* were synthesized under the same reaction conditions by replacing WO_3−_*_x_* with Ru-WO_3−_*_x_*. The CoO*_x_* loading amount was set at 1 wt%, 3 wt%, and 5 wt% (relative to the total mass) by varying the amount of added Co-EDTA-Na_2_.

**1.4 Preparation of Ru-WO_3−_*_x_*/CoO*_x_*-Loaded PVA Membranes**

PVA (375 mg) and PEG (30 mg) were dissolved in deionized water (5 mL) under ultrasonication, and glutaraldehyde (47.0 μL) was then added to this solution. The obtained mixture was named solution A. Simultaneously, the Ru-WO_3−_*_x_*/CoO*_x_* powder (25 mg) was dispersed into deionized water (296 μL). The obtained suspension was then mixed with dilute HNO_3_ (10 wt%, 204 μL) to give solution B. Solutions A and B were quickly mixed into a 50 mL beaker under ultrasonication. The mixture was heated in a water bath at 70 ℃ for 30 min to initiate polymerization, yielding a gel Ru-WO_3−_*_x_*/CoO*_x_*/PVA membrane. Liquid nitrogen was then added to freeze the gel membrane, followed by a freeze-drying process for 24 h to remove the water.

- 1. **Photocatalytic N_2_ Fixation Tests**

Photocatalytic N_2_ fixation experiments were carried out in a custom-built reactor equipped with a quartz window (PQ256, Beijing Perfectlight Technology Co., Ltd., window diameter: 6.5 cm; total volume: ~220 mL). The reactor was illuminated from the top using a Xe lamp (PLS-SXE300C, Beijing Perfectlight Technology Co., Ltd.) equipped with an AM 1.5G filter. The distance between the light source and the reactor was maintained at 30 cm. The reaction temperature was maintained at 20 °C by connecting the reactor to an external circulating water chiller. All glassware and the reactor were acid-washed and rinsed three times with deionized water, and the catalysts were pretreated and stored under vacuum to remove any adsorbed nitrogen-containing residues.

In a typical experiment, the catalyst (50 mg) was ultrasonically dispersed in deionized water (100 mL). High-purity N_2_ (99.99%) was then bubbled into the suspension at a flow rate of 50 mL min^−1^ through a gas inlet positioned below the liquid surface, while the solution was subjected under stirring at 500 rpm for 30 min to obtain an N_2_-saturated solution. The reactor was subsequently sealed, and the suspension was illuminated under simulated solar irradiation. The light intensity was measured using a radiometer (Meteon 2.0 irradiance meter, Kipp & Zonen) and calibrated to 100 mW cm^−2^ by adjusting the lamp current. At 30 min intervals, aliquots of the reaction solution were withdrawn with a syringe, and the suspended catalyst was removed by centrifugation and filtration. The concentrations of NH_4_^+^, NO_3_^−^, and NO_2_^−^ were determined by ion chromatography (SHINE CIC-D100, Qingdao Shenghan Chromatograph Technology).

The hydrazine concentration was quantified by the Watt–Chrisp method. Briefly, the reaction solution (1 mL) was mixed with the colorimetric reagent (5 mL, prepared by dissolving *p*-dimethylaminobenzaldehyde (5.99 g) into concentrated HCl (30 mL) and ethanol (300 mL)). The mixture was then stirred at room temperature for 10 min. The hydrazine yield was estimated from the absorbance of the resulting solution at 460 nm.^[1]^

The concentration of the produced NH_3_ was also cross-checked by the colorimetric method with Nessler’s reagent. Nessler’s reagent is composed of K_2_HgI_4_ (0.09 mol L^−1^), KOH (2.5 mol L^−1^), and deionized water. In a typical process, the analyte solution (0.5 mL) was first mixed with KNaC_4_H_4_O_6_ solution (0.2 mol L^−1^, 0.25 mL) to minimize the interference of other ions. Nessler’s reagent (0.25 mL) was then added to the mixture, and the entire mixture was left undisturbed for 15 min. The NH_3_ concentration was determined by monitoring the absorbance at 425 nm.^[2]^

**1.6 AQE and SCCE Determination**

The photocatalytic tests for determining the apparent quantum efficiencies (AQEs) and solar-to-chemical conversion efficiencies (SCCEs) were conducted with a custom-built cylinder reactor. For the measurement of AQEs, the AM 1.5G light filter was replaced with monochromatic bandpass filters of different wavelengths (350 nm, 380 nm, 420 nm, 475 nm, 520 nm, 550 nm, 600 nm, 650 nm, 700 nm). For measuring the AQEs at 808 nm and 980 nm, lasers were used as light sources, and the photocatalytic tests were conducted in a small cuvette. The AQEs were estimated according to the following equation

$\mathrm{AQE}=\frac{N_{e}}{N_{p}}=\frac{n\times N_{NH_{3}}}{\frac{P\times S\times t}{h\nu}}\times100\%$ (1)

where *N*_e_, *N*_p_, and $N_{NH_{3}}$ represent the numbers of reacted electrons, incident photons and generated NH_3_ molecules, respectively; *n* is the number of electrons transferred in the reaction (3 for NH_3_ production); ℎ is the Planck constant; *P*, *S*, and *t* represent the incident light intensity, solar irradiation area, and reaction time, respectively.

The SCCE was determined according to the following equation

$\mathrm{SCCE}=\frac{\Delta G\times n}{P\times S\times t}\times100\%$ (2)

where *∆G* is the change in the Gibbs free energy (339.0 kJ mol^−1^ for NH_3_ production),^[2]^ *n* is the amount of NH_3_ generated in the reaction; *P*, *S*, and *t* denote the incident light intensity, solar irradiation area, and reaction time, respectively.

**1.7 Photoelectrochemical Experiments**

All electrochemical measurements were performed on a standard electrochemical workstation (CHI 760E, Shanghai Chenhua Instruments) with a three-electrode system. The working electrode was prepared by dropping the dispersion of the photocatalyst sample in an ethanolic Nafion solution onto a 1 cm × 1 cm fluorine-doped tin oxide-coated glass substrate. A standard Ag/AgCl electrode and a 1 cm × 1 cm platinum foil were utilized as the reference electrode and counter electrode, respectively. The electrolyte solution was a 0.1 M Na_2_SO_4_ solution. Mott–Schottky plots were obtained using the impedance-potential technique to calculate the free electron density. The Mott-Schottky equation is given below

$\frac{1}{C^{2}}=\frac{2}{\left( e\varepsilon_{0}\varepsilon_{r}NA^{2} \right)}{\times(E-E}_{\mathrm{fb}}-\frac{k_{B}T}{e})$ (3)

where *C* represents the interfacial capacitance, *e* represents the elementary charge, *ε*_0_ is the vacuum permittivity, *ε*_r_ is the relative permittivity of the electrode material (*ε*_r_ = 20 for WO_3_),^[3]^ *N* represents the charge carrier density, *A* represents the area of the electrode, *E* denotes the applied voltage, *E*_fb_ represents the flatband potential, *k*_B_ is the Boltzmann constant, and *T* is the temperature in Kelvin. *N* can be obtained from the slope of the straight-line part of the curve by plotting 1/*C*^2^ against *E*.

The photocurrent was measured by chronoamperometry with no bias potential applied. The light source was the same as the photocatalytic experiments and was turned on and off every 30 s. Before the measurement, the electrolyte was saturated with Ar or N_2_ atmosphere for 30 min.

The electrochemical impedance spectra (EIS) were measured using the alternate-current impedance technique. The frequency range was set at 0.01–100000 Hz.

**1.8 ^15^N_2_ Isotope Labelling Experiments**

Isotopic labeling experiments were performed by replacing ^14^N_2_ with ^15^N_2_ as the feed gas during photocatalysis. After the reaction, the reaction solution (10 mL) was first filtered through a 0.22 μm membrane to remove the solid catalyst. The pH of the filtrate was then adjusted to 2 using HCl (1 mol L^−1^), which was followed by concentrating the solution through rotary evaporation at a low temperature. Finally, DMSO-d_6_ (100 μL) was mixed with the concentrated solution (500 μL), and the resulting mixture was analyzed by ^1^H NMR.

**1.9 NH_4_^+^ Adsorption–Desorption Experiments**

The catalyst (50 mg) was dispersed in an aqueous NH_4_Cl solution (1 g L^−1^, 50 mL), which was followed by stirring in the dark for 2 h to allow adsorption equilibrium. The catalyst was then collected by centrifugation and redispersed in pure water (50 mL). The suspension was continuously bubbled with Ar gas and illuminated by a 300 W Xenon lamp at 100 mW cm^−2^. The concentration of NH_4_^+^ in the solution was determined by ion chromatography to quantify the NH_4_^+^ desorption rate.

**1.10 DFT Calculations**

All first-principles calculations were performed based on the density functional theory (DFT), which was implemented in the Vienna Ab-initio Simulation Package (VASP).^[4–6]^ The projector-augmented wave (PAW) pseudo-potential method and plane-wave basis set were employed to describe the ionic cores and to take valence electrons into account, respectively.^[7,8]^ The configurations 5*p*^6^5*d*^4^6*s*^2^ of W, 4*p*^6^4*d*^7^5*s*^1^ of Ru, 3*d*^7^4*s*^2^ of Co, 2*s*^2^2*p*^3^ of N, and 2*s*^2^2*p*^4^ of O were treated as valence electrons for the PAW pseudo-potentials. To ensure high numerical accuracy, an energy cutoff of 550 eV was used for the expansion of the plane-wave basis set, which is higher than the typical range (400–450 eV) employed in related studies to guarantee excellent convergence.^[9,10]^ The generalized-gradient approximation (GGA) of Perdew-Burke-Ernzerhof (PBE) was employed for the electronic exchange-correlation functional.^[11]^ No Hubbard U correction was applied, as this approach has been validated for reliably describing relative reaction trends on WO_3_ surfaces in prior studies and aligns with the comparative focus of our work.^[9,12]^

The surface was modeled using a periodic slab approach. Based on the WO_3_ (200) facet, a 2 × 2 supercell was constructed, yielding lateral dimensions of approximately 15.5 Å to eliminate spurious interactions between the periodic images of adsorbates. The slab comprised of three W–O atomic layers (~11.6 Å thick), with the bottommost layer fixed at its bulk positions to simulate a semi-infinite substrate. A vacuum space of exceeding 15 Å between two layers was added to avoid interactions between periodic images.

All structures investigated in this study were fully relaxed until the Hellmann-Feynman forces acting on all atoms were less than 0.05 eV Å^−1^, and the total energy was smaller than 1 × 10^−5^ eV. The *k*-point sampling was obtained from the Γ-centered scheme with a 3 × 3 × 1 mesh for the slab model, which corresponds to a fine *k*-spacing of ~0.12 Å^−1^ and exceeds common empirical thresholds for accuracy.^[13,14]^

The adsorption energy (*E*_ads_) of N_2_ on the surface was calculated using the formula

$$E_{\mathrm{ads}}=E_{N_{2}^{*}}-E_{\mathrm{slab}}-E_{N_{2}}$$

where $E_{\mathrm{ads}}$ is the adsorption energy, $E_{N_{2}^{*}}$ is the energy of N_2_ adsorbed on the slab, $E_{\mathrm{slab}}$ is the energy of the slab before N_2_ adsorption, and $E_{N_{2}}$ is the energy of a free N_2_ molecule.

The Crystal Orbital Hamiltonian Population (COHP) was calculated using the LOBSTER package.^[15]^ The Bader charge analysis method was used to characterize the charge state and charge transfer.^[16,17]^

**1.11 Statistical Analysis**

Unless otherwise stated, all photocatalytic measurements were performed in at least three independent experiments using separately prepared samples. Raw data were first inspected for consistency, and clear outliers attributable to identifiable experimental errors (e.g., instrumental malfunction, leakage, or sampling mistakes) were excluded. Unless otherwise stated, none of raw data has undergone any artificial preprocessing. Data are presented as mean ± standard deviation (SD). For comparisons between two groups, statistical significance was assessed using a two-tailed unpaired Student’s t-test. For comparisons among more than two groups, a one-way analysis of variance (ANOVA) followed by a Tukey post hoc test was employed. Significance level of *α* = 0.05 was used throughout the study.

All statistical analyses were carried out using OriginPro 2023 (OriginLab) and Igor 9.0 (WaveMetrics), and plotting was performed with the same software packages.

1. **Supplementary Figures**


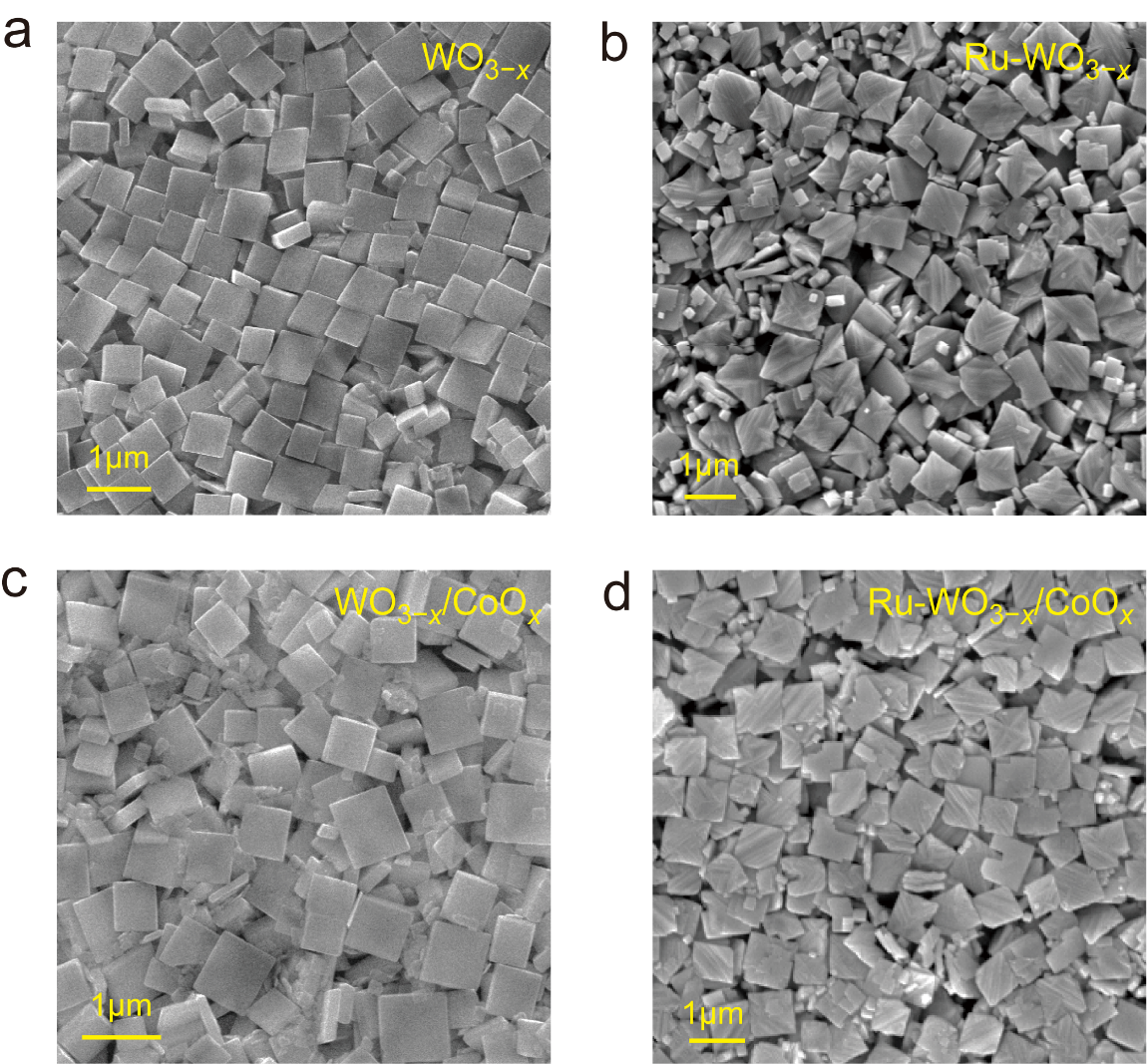


**Figure S1.** SEM images. a) WO_3−_*_x_*. b) Ru-WO_3−_*_x_*. c) WO_3−_*_x_*/CoO*_x_*. d) Ru-WO_3−_*_x_*/CoO*_x_*.


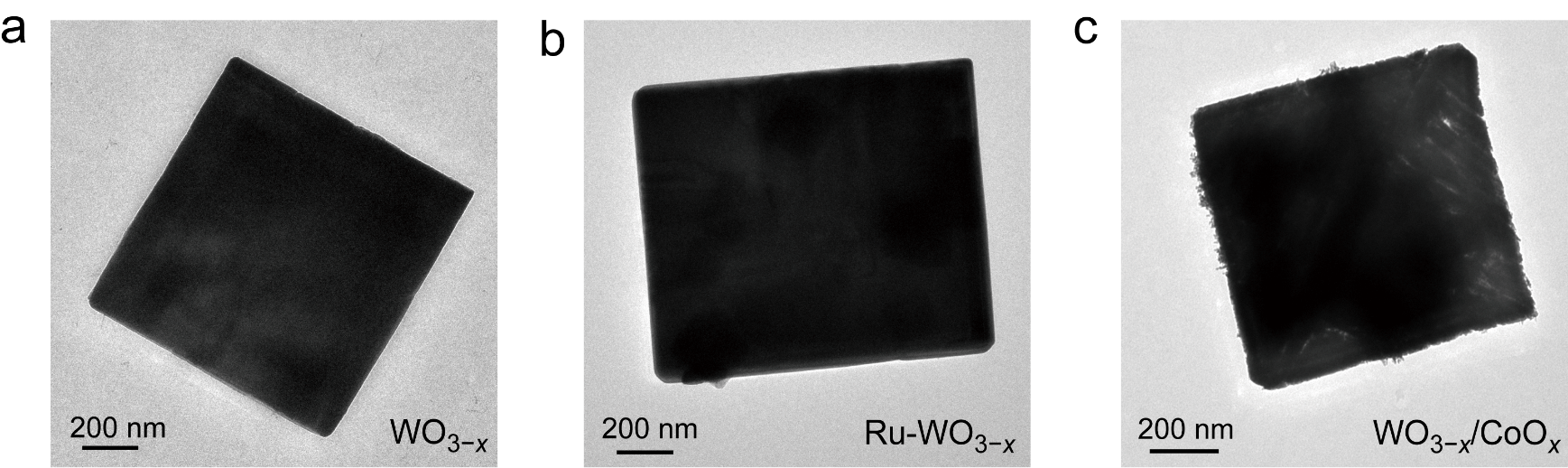


**Figure S2.** TEM images. a) WO_3−_*_x_*. b) Ru-WO_3−_*_x_*. c) WO_3−_*_x_*/CoO*_x_*.


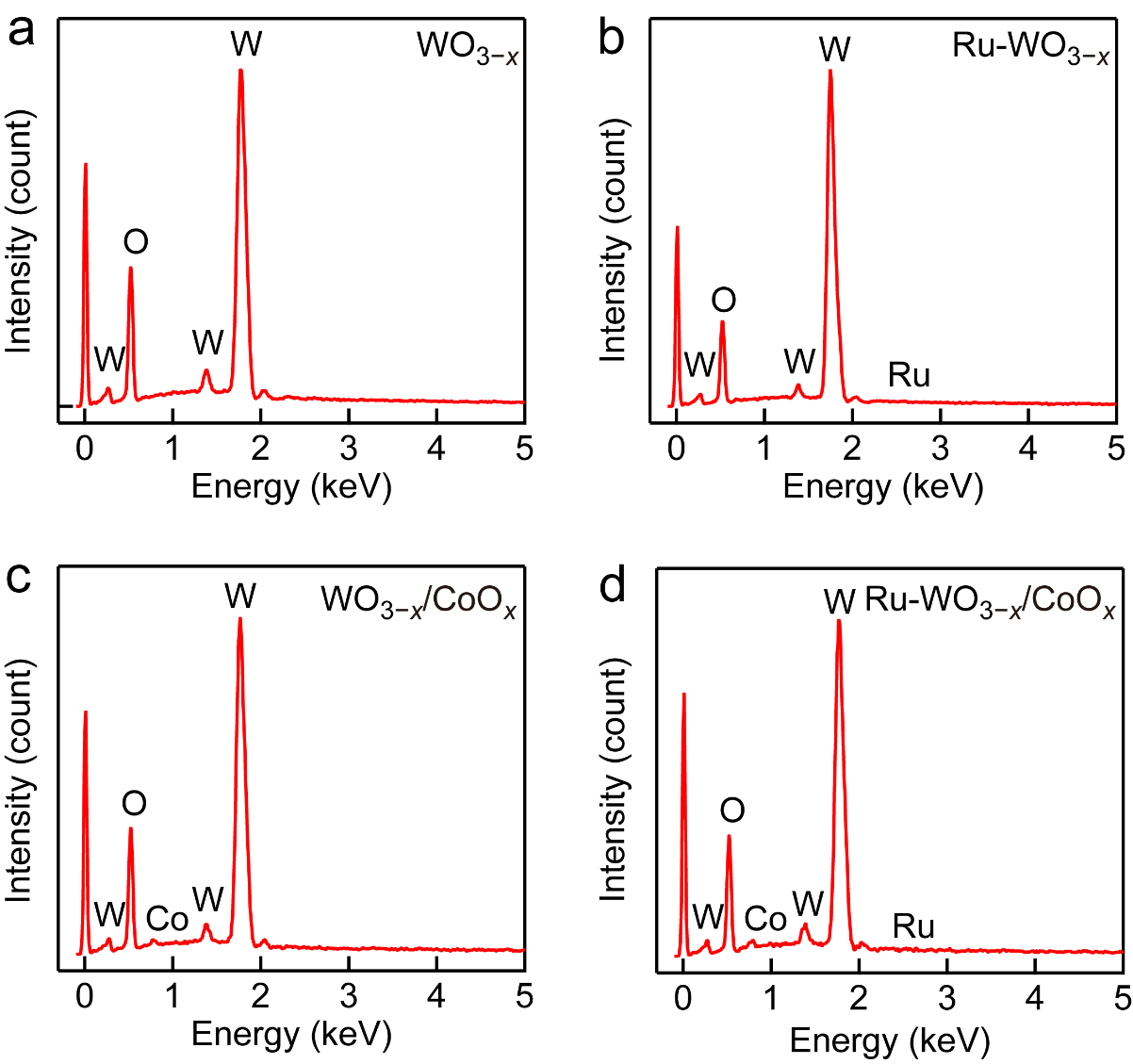


**Figure S3.** EDX spectra. a) WO_3−_*_x_*. b) Ru-WO_3−_*_x_*. c) WO_3−_*_x_*/CoO*_x_*. d) Ru-WO_3−_*_x_*/CoO*_x_*.


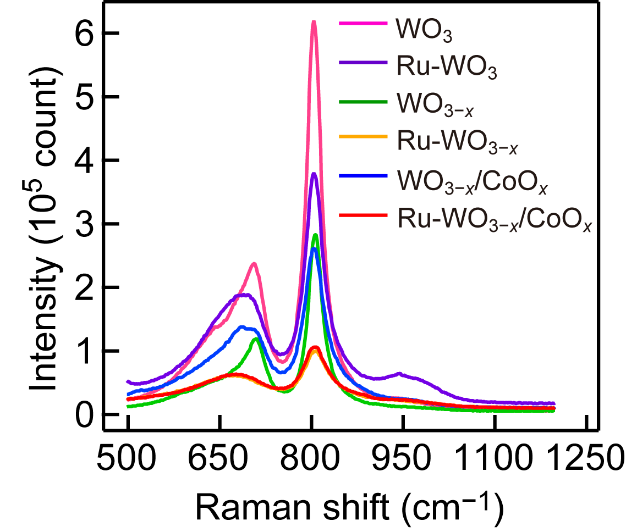


**Figure S4.** Raman spectra of the WO_3_-based samples.


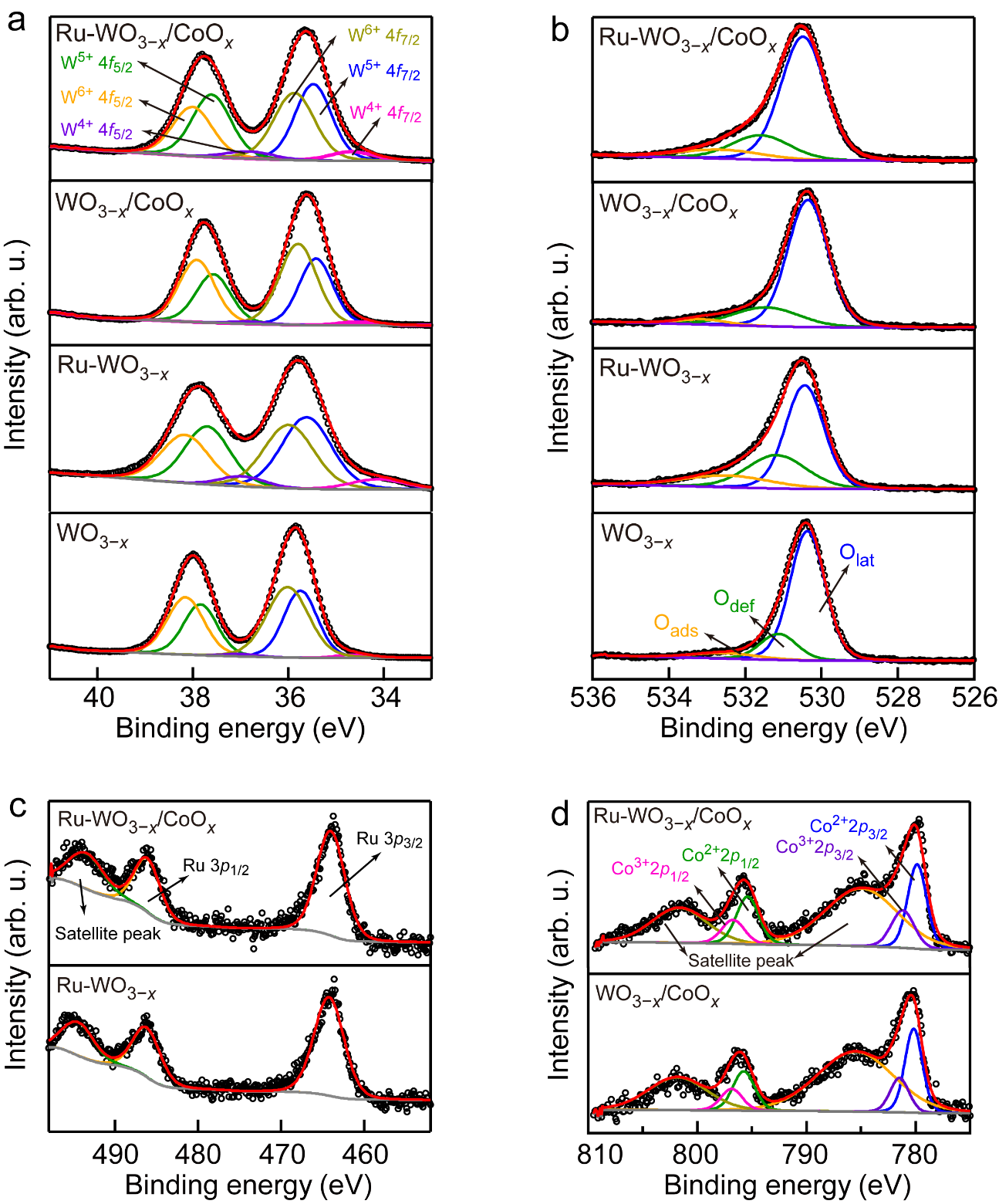


**Figure S5.** XPS spectra. a) W 4*f*. b) O 1*s*. c) Ru 3*p*. d) Co 2*p*.

**Discussion**

The W 4*f* XPS spectra of WO_3−_*_x_* can be divided into the contributions from three species, including W^6+^ (36.0 eV and 38.2 eV), W^5+^ (35.7 eV and 37.8 eV), and W^4+^ (34.7 eV and 37.0 eV). The existence of the low-valence state W originates from the introduction of OVs. The peak shape and peak position of other samples showed no significant change compared to WO_3−_*_x_*. In the O 1*s* XPS spectra, three deconvoluted peaks are displayed. The peaks centered at 530.4 eV, 531.4 eV, and 532.3 eV can be ascribed to the lattice oxygen (O_lat_), oxygen defect (O_def_), and adsorbed oxygen (O_ads_), respectively. The peak area of O_def_ is much larger in the Ru-doped samples, proving that Ru doping can induce additional OVs in the materials. The Ru 3*p* XPS spectra of Ru-WO_3−_*_x_* and Ru-WO_3−_*_x_*/CoO*_x_* show two broad peaks at ~464.2 eV and ~486.2 eV, which are attributed to Ru 3*p*_3/2_ and Ru 3*p*_1/2_ of Ru*^n^*^+^ species, respectively. The absence of a distinct metallic Ru^0^ peak (typically observed at ~462 eV and ~484 eV) indicates that Ru is predominantly present in oxidized states within the lattice, with only a negligible metallic contribution.^[18]^ The Co 2*p* XPS spectra show the mixed oxidation states of Co in the sample, indicating that CoO*_x_* exists as a mixed phase rather than a single phase. Semi-quantitative analysis shows a Co^2+^/Co^3+^ ratio of approximately 2:1, suggesting that Co is present predominantly as Co^2+^ (CoO), accompanied by a minor fraction of Co^3+^ (Co_3_O_4_/CoOOH).^[19]^

The detailed fitting parameters for each XPS spectrum are shown in Tables S2–S4.


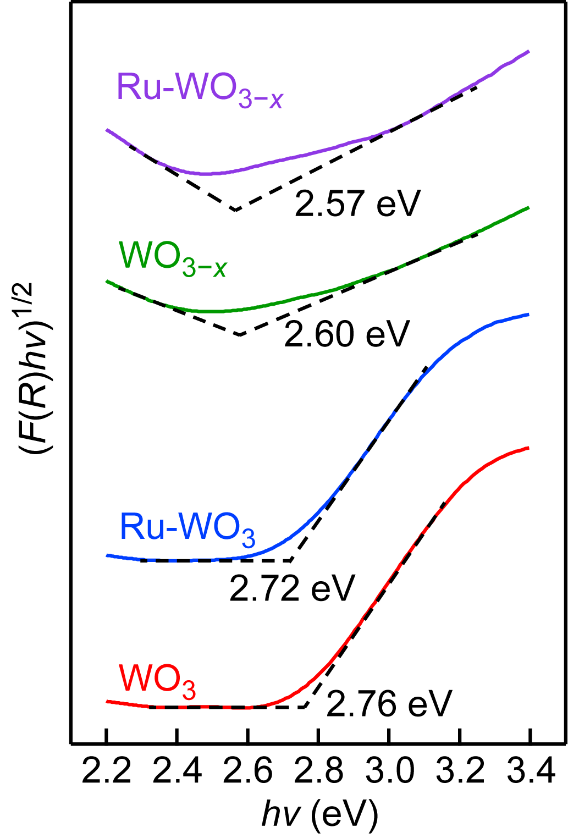


**Figure S6.** Tauc plots showing the bandgaps of WO_3_, Ru-WO_3_, WO_3−_*_x_*, and Ru-WO_3−_*_x_*. *F*(*R*) is the Kubelka-Munk function

$F\left( R \right)=\frac{{(1-R)}^{2}}{2R}$ (4)

where *R* represents the reflectance of the sample measured by ultraviolet–visible diffuse reflectance spectroscopy.


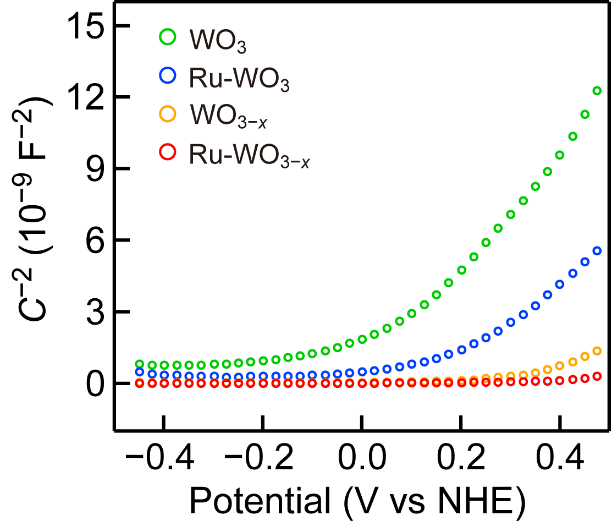


**Figure S7.** Mott–Schottky plots of WO_3_, Ru-WO_3_, WO_3−_*_x_*, and Ru-WO_3−_*_x_*.


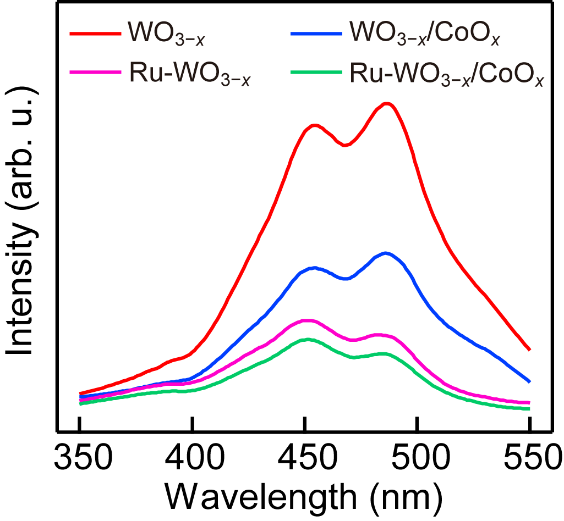


**Figure S8.** Photoluminescence spectra of WO_3−_*_x_*, Ru-WO_3−_*_x_*, WO_3−_*_x_*/CoO*_x_*, and Ru-WO_3−_*_x_*/CoO*_x_*.


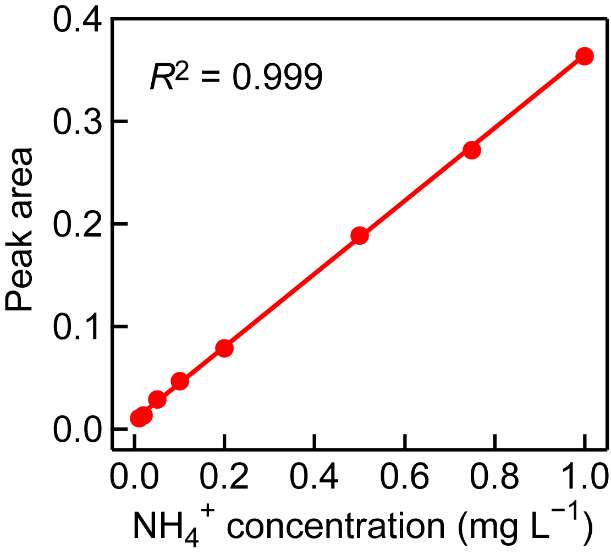


**Figure S9.** Standard calibration curve for determining the ammonia concentration by ion chromatography.


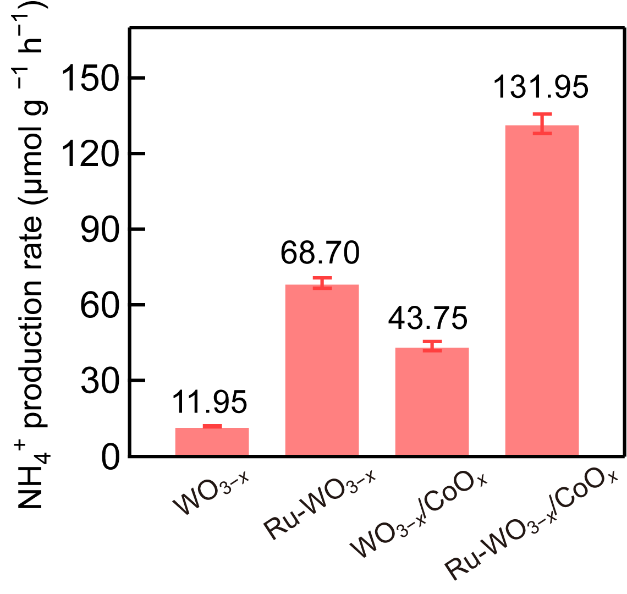


**Figure S10.** NH_3_ production rates of WO_3−_*_x_*, Ru-WO_3−_*_x_*, WO_3−_*_x_*/CoO*_x_*, and Ru-WO_3−_*_x_*/CoO*_x_*.


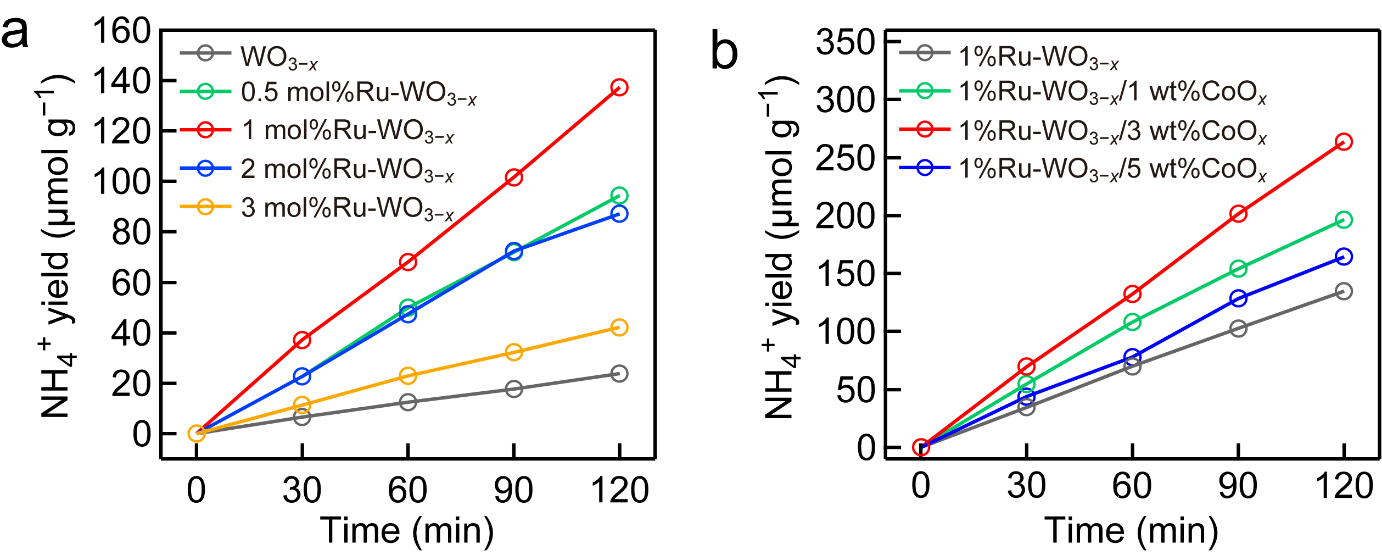


**Figure S11.** Optimization of the Ru doping amount and CoO*_x_* loading amount. a) Time-dependent NH_3_ yields of Ru-WO_3−_*_x_* with different molar ratios of Ru. b) Time-dependent NH_3_ yields of 1% Ru-WO_3−_*_x_*/CoO*_x_* with different weight ratios of CoO*_x_*.


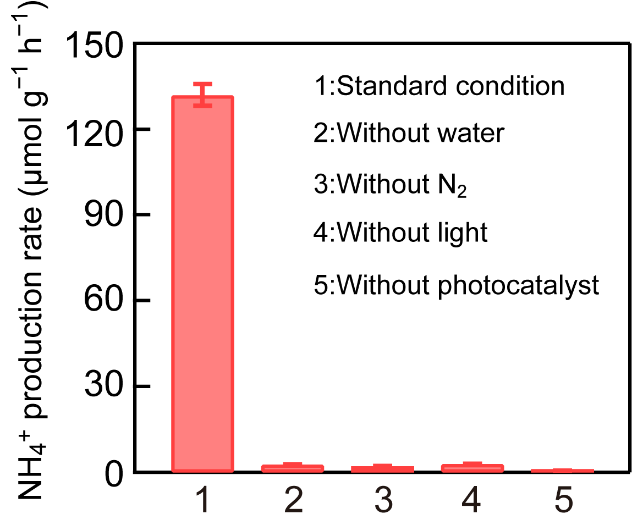


**Figure S12.** NH_3_ production rates of Ru-WO_3−_*_x_*/CoO*_x_* under different control experimental conditions.


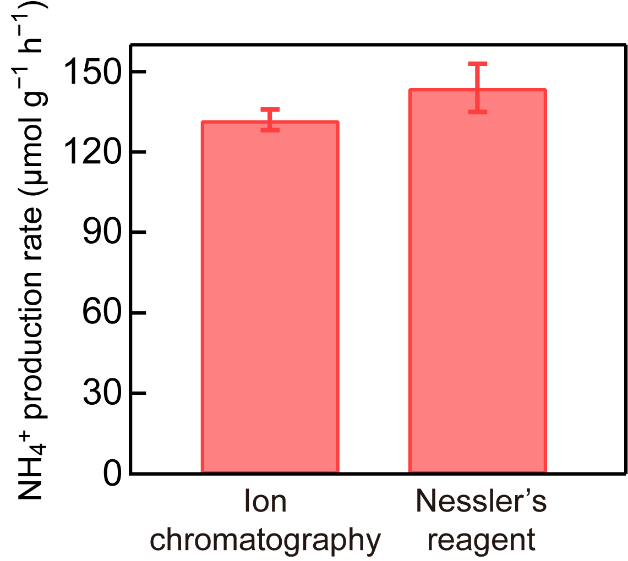


**Figure S13.** Comparison of the NH_3_ production rates of Ru-WO_3−_*_x_*/CoO*_x_* determined by ion chromatography and the Nessler’s reagent method.


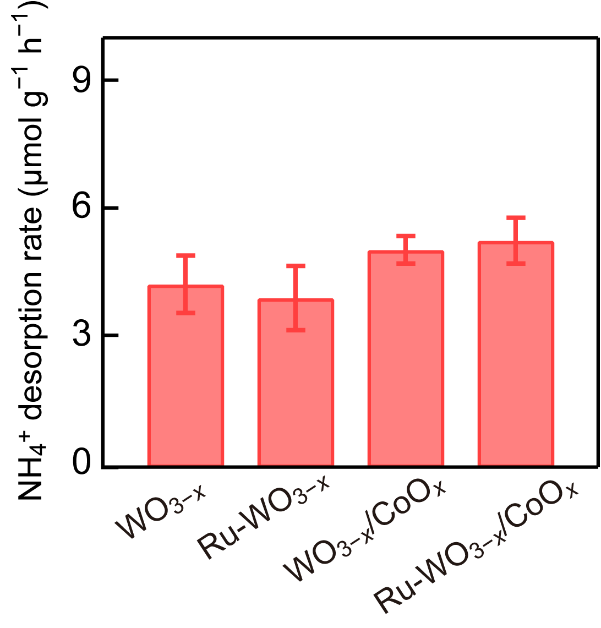


**Figure S14.** Negligible NH_4_^+^ desorption rates of pre-equilibrated WO_3−_*_x_*, Ru-WO_3−_*_x_*, WO_3−_*_x_*/CoO*_x_*, and Ru-WO_3−_*_x_*/CoO*_x_* in pure water.


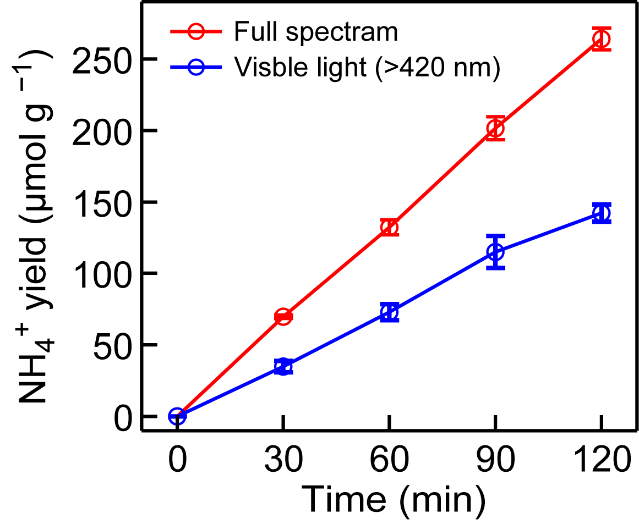


**Figure S15.** Time-dependent NH_3_ yields of Ru-WO_3−_*_x_*/CoO*_x_* under different light illumination.


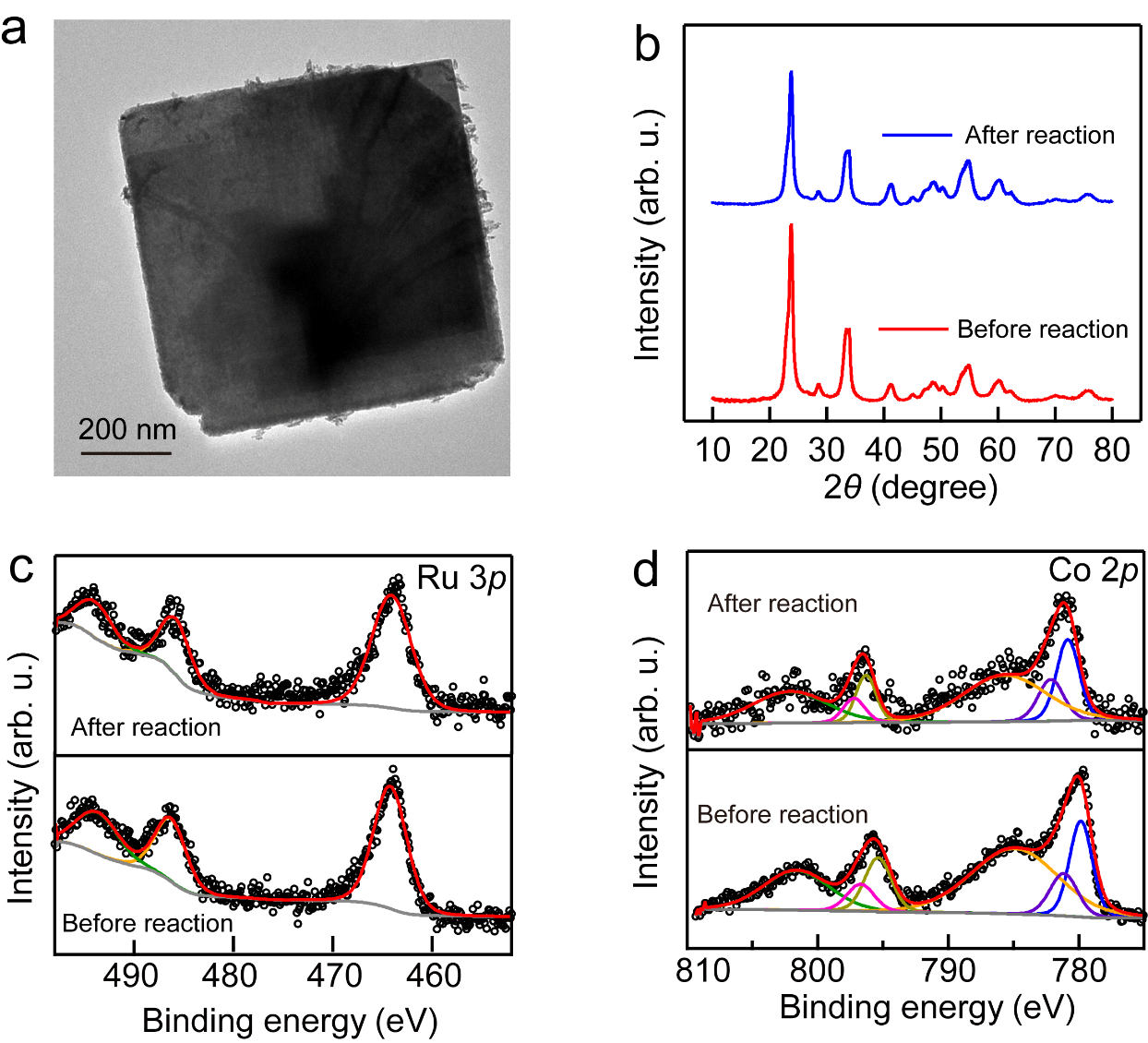


**Figure S16.** Stability evaluation of Ru-WO_3−_*_x_*/CoO*_x_*. a) TEM image of Ru-WO_3−_*_x_*/CoO*_x_* after the cycling experiment. b) XRD patterns of Ru-WO_3−_*_x_*/CoO*_x_* before and after the cycling experiment. c) Ru 3*p* XPS spectra of Ru-WO_3−_*_x_*/CoO*_x_* before and after the cycling experiment. d) Co 2*p* XPS spectra of Ru-WO_3−_*_x_*/CoO*_x_* before and after the cycling experiment.


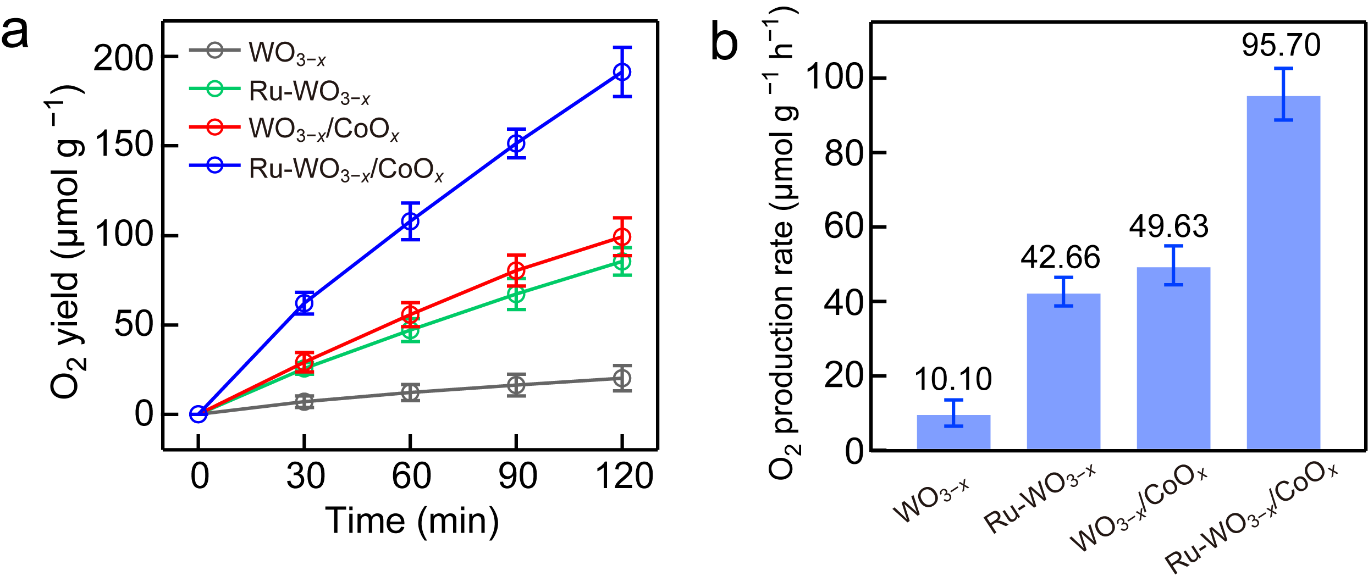


**Figure S17.** O_2_ evolution performance. a,b) Time-dependent O_2_ yields (a) and O_2_ production rates (b) of WO_3−_*_x_*, Ru-WO_3−_*_x_*, WO_3−_*_x_*/CoO*_x_*, and Ru-WO_3−_*_x_*/CoO*_x_*.


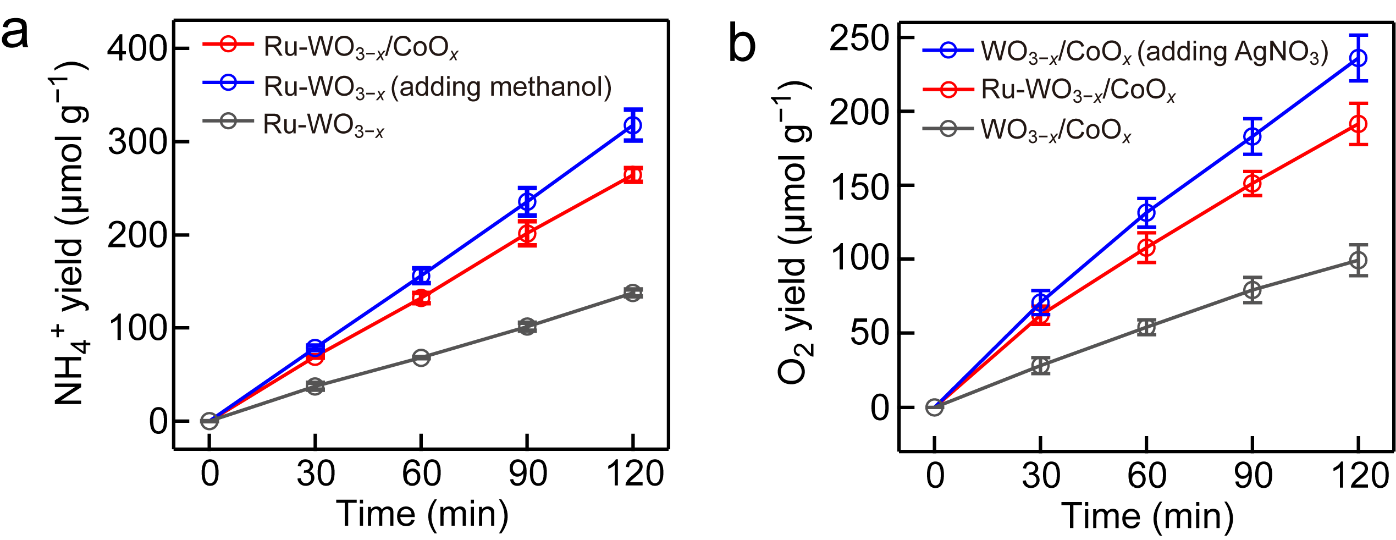


**Figure S18.** Control experiments by adding sacrificial agents. a) Comparison of the improvement in the NH_3_ synthesis performance by adding a sacrificial agent for holes and introducing the oxidation active sites. b) Comparison of the enhancement in the O_2_ evolution reaction by adding a sacrificial agent for electrons and introducing the reduction active sites.


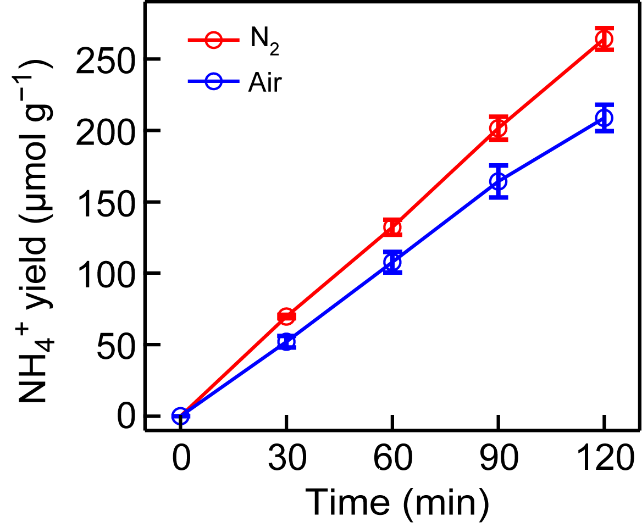


**Figure S19.** Comparison of the time-dependent NH_3_ yields of Ru-WO_3−_*_x_*/CoO*_x_* under N_2_ and air atmosphere.


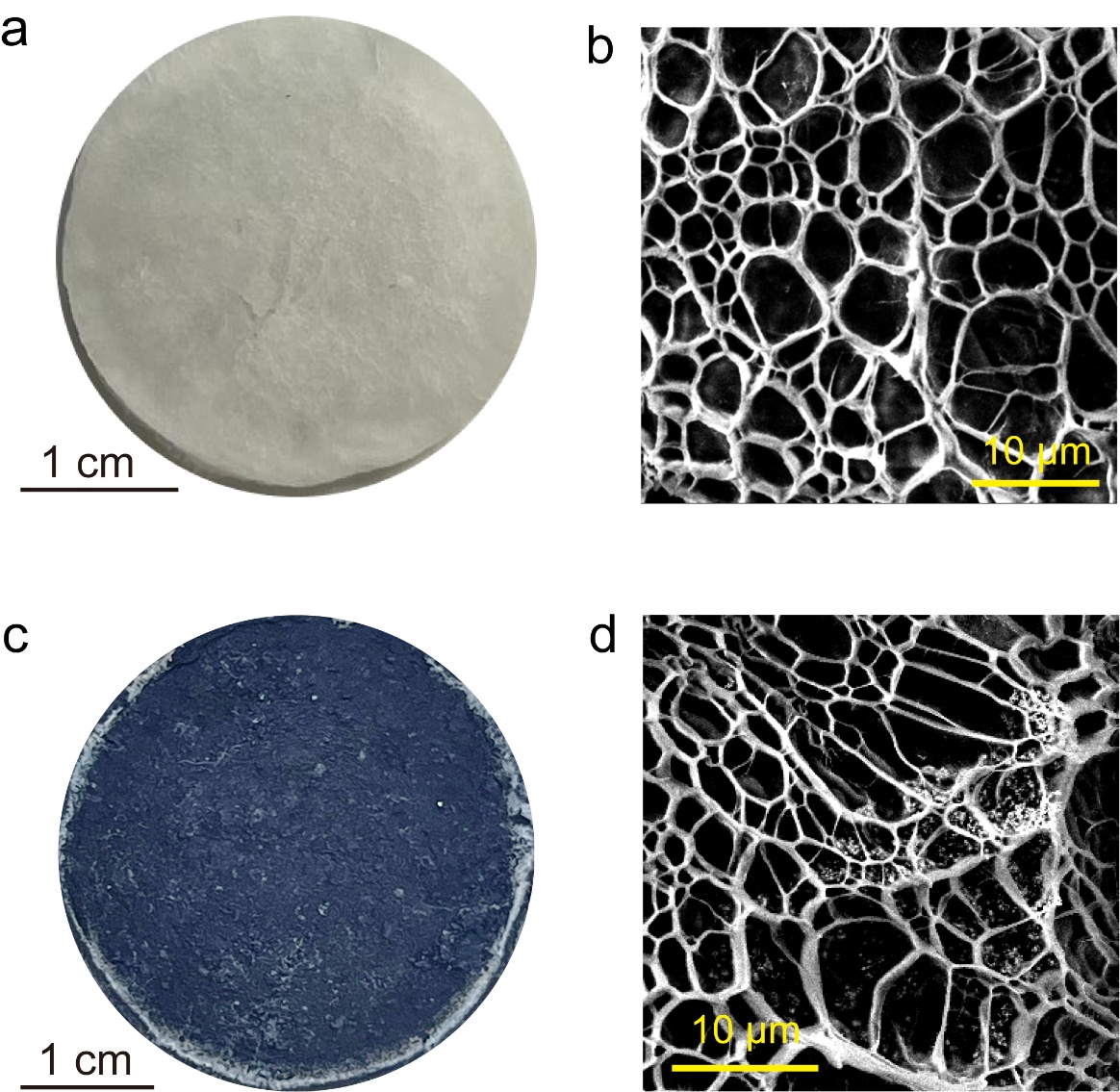


**Figure S20.** Characterization of the membranes. a,b) Photograph (a) and SEM image (b) of a PVA membrane. c,d) Photograph (c) and SEM image (d) of a PVA-supported Ru-WO_3−_*_x_*/CoO*_x_* photocatalytic membrane.


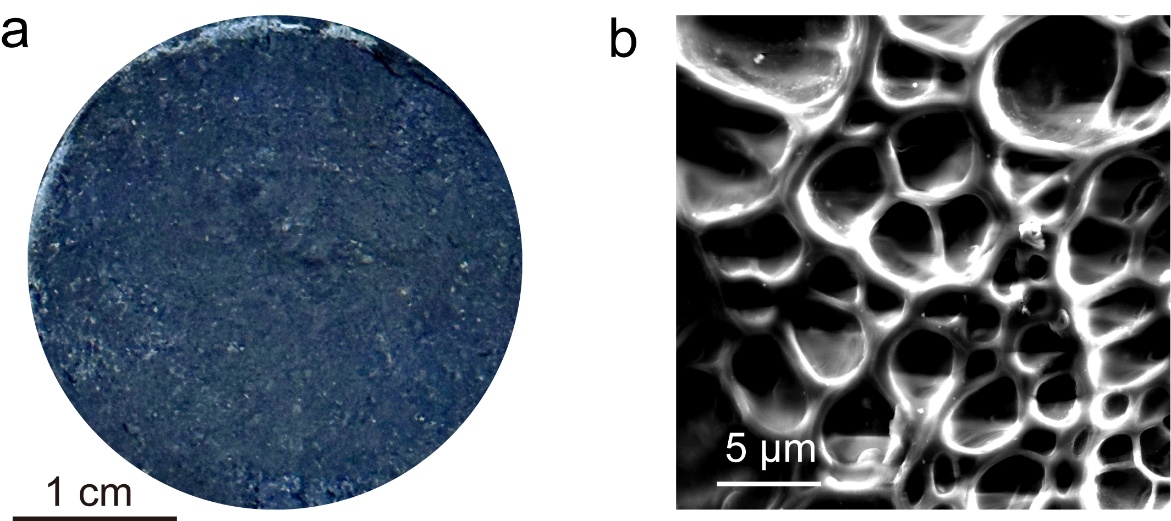


**Figure S21.** Characterization of the Ru-WO_3−_*_x_*/CoO*_x_* photocatalytic membrane after the cycling experiment. a) Photograph. b) SEM image.


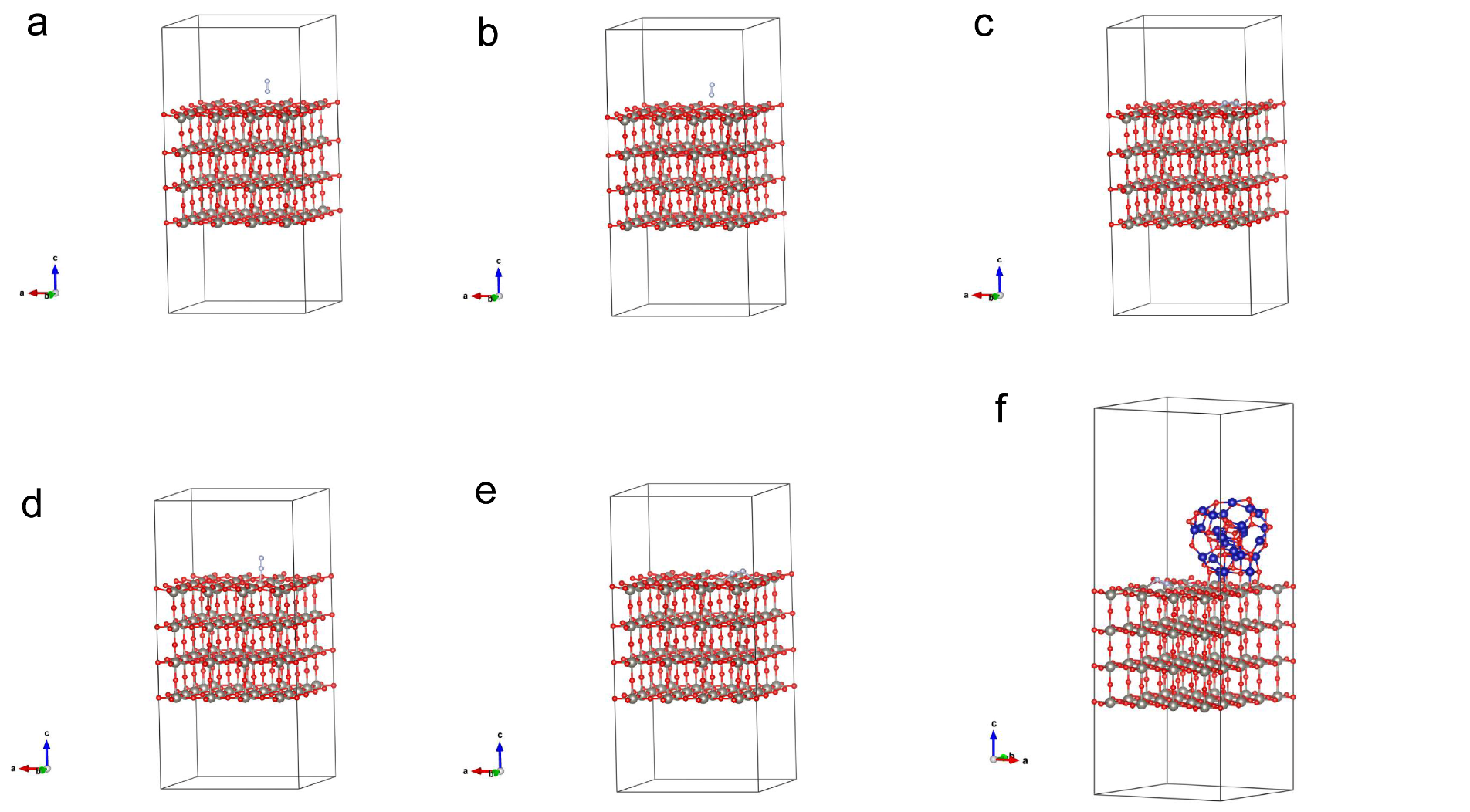


**Figure S22.** Structure models for DFT calculations. a) Free N_2_ and WO_3_. b) N_2_ adsorbed on WO_3−_*_x_* by the end-on mode. c) N_2_ adsorbed on WO_3−_*_x_* by the side-on mode. d) N_2_ adsorbed on Ru-WO_3−_*_x_* by the end-on mode. e) N_2_ adsorbed on Ru-WO_3−_*_x_* by the side-on mode. f) N_2_ adsorbed on Ru-WO_3−_*_x_*/CoO*_x_* by the side-on mode.

**3 Supplementary Tables**

**Table S1.** EDX analysis results of element compositions in the different samples.

| Sample | W (at%) | O (at%) | W:O | Ru (at%) | Ru:W | Co (at%) | Co:W |
| --- | --- | --- | --- | --- | --- | --- | --- |
| WO_3_ | 26.28 | 73.72 | 1:2.81 | / | / | / | / |
| Ru-WO_3_ | 27.92 | 71.74 | 1:2.57 | 0.34 | 0.012:1 | / | / |
| WO_3−_*_x_* | 29.86 | 70.14 | 1:2.35 | / | / | / | / |
| Ru-WO_3−_*_x_* | 31.31 | 68.41 | 1:2.19 | 0.28 | 0.009:1 | / | / |
| WO_3−_*_x_*/CoO*_x_* | 28.89 | 68.80 | 1:2.38 | / | / | 2.31 | 0.080:1 |
| Ru-WO_3−_*_x_*/CoO*_x_* | 30.55 | 66.43 | 1:2.18 | 0.26 | 0.009:1 | 2.76 | 0.089:1 |

**Table S2.** Fitting parameters of the W 4*f* XPS spectra.

| Sample | | WO_3−_*_x_* | Ru-WO_3−_*_x_* | WO_3−_*_x_*/CoO*_x_* | Ru-WO_3−_*_x_*/CoO*_x_* |
| --- | --- | --- | --- | --- | --- |
| W^6+^ (4*f*_7/2_) | FWHM | 1.035 | 1.237 | 0.925 | 1.008 |
|  | Binding energy (eV) | 36.01 | 35.98 | 35.79 | 35.88 |
| W^5+^ (4*f*_7/2_) | FWHM | 0.838 | 1.223 | 0.838 | 0.916 |
|  | Binding energy (eV) | 35.71 | 35.63 | 35.42 | 35.48 |
| W^4+^ (4*f*_7/2_) | FWHM | 0.830 | 1.332 | 0.897 | 1.076 |
|  | Binding energy (eV) | 34.66 | 34.22 | 34.56 | 34.58 |
| W^6+^ (4*f*_5/2_) | FWHM | 0.907 | 1.179 | 0.883 | 0.975 |
|  | Binding energy (eV) | 38.15 | 38.16 | 37.91 | 38.01 |
| W^5+^ (4*f*_5/2_) | FWHM | 0.833 | 1.115 | 0.868 | 0.955 |
|  | Binding energy (eV) | 37.84 | 37.70 | 37.57 | 37.62 |
| W^4+^ (4*f*_5/2_) | FWHM | 0.826 | 1.088 | 0.925 | 1.167 |
|  | Binding energy (eV) | 36.98 | 36.96 | 36.82 | 36.82 |
| W^6+^ ratio | | 53.08% | 43.68% | 56.05% | 46.31% |
| W^5+^ ratio | | 42.49% | 48.81% | 41.67% | 47.01% |
| W^4+^ ratio | | 4.43% | 7.51% | 2.28% | 6.67% |

**Table S3.** Fitting parameters of THE Ru 3*p* XPS spectra.

| Sample | | Ru-WO_3−_*_x_* | Ru-WO_3−_*_x_*/CoO*_x_* |
| --- | --- | --- | --- |
| Ru*^n^*^+^ (3*p*_3/2_) | FWHM | 3.860 | 3.798 |
|  | Binding energy (eV) | 464.25 | 464.20 |
| Ru*^n^*^+^ (3*p*_1/2_) | FWHM | 3.949 | 3.693 |
|  | Binding energy (eV) | 486.17 | 486.29 |

**Table S4.** Fitting parameters of the Co 2*p* XPS spectra.

| Sample | | WO_3−_*_x_*/CoO*_x_* | Ru-WO_3−_*_x_*/CoO*_x_* |
| --- | --- | --- | --- |
| Co^3+^ (2*p*_3/2_) | FWHM | 2.027 | 2.2937 |
|  | binding energy (eV) | 781.47 | 781.25 |
| Co^2+^ (2*p*_3/2_) | FWHM | 1.893 | 2.048 |
|  | binding energy (eV) | 780.17 | 779.83 |
| Co^3+^ (2*p*_1/2_) | FWHM | 2.301 | 2.485 |
|  | binding energy (eV) | 796.86 | 796.71 |
| Co^2+^ (2*p*_1/2_) | FWHM | 2.316 | 2.418 |
|  | binding energy (eV) | 795.74 | 795.37 |
| Co^3+^ ratio | | 30.53% | 32.79% |
| Co^2+^ ratio | | 69.47% | 67.21% |

**Table S5.** Estimated charge carrier densities of the different samples.

| Sample | Charge carrier density (cm^−3^) |
| --- | --- |
| WO_3_ | 1.52 × 10^20^ |
| Ru-WO_3_ | 3.27 × 10^20^ |
| WO_3−_*_x_* | 1.24 × 10^21^ |
| Ru-WO_3−_*_x_* | 2.38 × 10^21^ |

**Table S6.** Fitting results for the TRPL spectra of the different photocatalysts.

| Sample | WO_3−_*_x_* | Ru-WO_3−_*_x_* | WO_3−_*_x_*/CoO*_x_* | Ru-WO_3−_*_x_*/CoO*_x_* |
| --- | --- | --- | --- | --- |
| A_1_ (%) | 40.63 | 42.37 | 44.88 | 46.02 |
| *τ*_1_ (ns) | 1.390 | 2.160 | 2.713 | 3.989 |
| A_2_ (%) | 59.37 | 57.63 | 55.12 | 53.98 |
| *τ*_2_ (ns) | 5.391 | 14.172 | 17.976 | 21.937 |
| *τ*_ave_ (ns) | 4.791 | 12.962 | 16.305 | 19.528 |

The data were fitted using a bi-exponential function

$I\left( t \right)=A_{1}e^{-\frac{t}{\tau_{1}}}+A_{2}e^{-\frac{t}{\tau_{2}}}$ (5)

The intensity-weighted average lifetime *τ*_ave_ was calculated as below

$\tau_{\mathrm{ave}}=\frac{A_{1}\tau_{1}^{2}+A_{2}\tau_{2}^{2}}{A_{1}\tau_{1}+A_{2}\tau_{2}}$ (6)

**Table S7.** Photocatalytic nitrogen fixation performances of reported WO_3_-based materials.

| Photocatalyst | Light source | Light intensity | Quantification method | Sacrificial agent | NH_3_ yield  (μmol g_cat_^−1^ h^−1^) | Reference |
| --- | --- | --- | --- | --- | --- | --- |
| Mo-W_18_O_49_ | 300 W Xe lamp  (full spectrum) | 200 mW cm^−2^ | Ion chromatography | Na_2_SO_3_ | 195.5 | [20] |
|  |  |  |  |  |  |  |
| Mn-WO_3_ | 300 W Xe lamp  (full spectrum) | 250 mW cm^−2^ | Nessler’s reagent method | / | 425 | [21] |
| Fe-SA/WO_2.72–_*_x_* | 300 W Xe lamp  (full spectrum) | / | Ammonia ion-selective electrode | / | 186.5 | [22] |
| BPQDs/Fe-W_18_O_49_ | 300 W Xe lamp  (full spectrum) | 100 mW cm^−2^ | Ion chromatography | Na_2_SO_3_ | 187.6 | [23] |
| Au/Mo-W_18_O_49_ | 300 W Xe lamp  (full spectrum) | / | Nessler’s reagent method | methanol | 399.24 | [24] |
| CdS/WO_3_ | 300 W Xe lamp  (full spectrum) | / | Ion chromatography | / | 35.8 | [25] |
| WO_3_/C(CN)_3_ | 300 W Xe lamp  (*λ*>420 nm) | / | Nessler’s reagent method | methanol | 20.5 | [26] |
| MoO_3–_*_x_*/Fe-W_18_O_49_ | 300 W Xe lamp  (full spectrum) | 200 mW cm^−2^ | Indophenol blue method | / | 137.5 | [27] |
| Ru/W_18_O_49_ | 300 W Xe lamp  (full spectrum) | / | Nessler’s reagent method | / | 44.3 | [28] |
| WO_3–_*_x_* nanosheets | LED lamp  (4 × 3 W) | / | Nessler’s reagent method | Na_2_SO_3_ | 82.41 | [29] |
| Sb_2_O_3_/W_18_O_49_ | 300 W Xe lamp  (*λ*>420 nm) | / | Indophenol blue method | methanol | 35.3 | [30] |
| Cu_2_O/W_18_O_49_ | 300 W Xe lamp  (full spectrum) | / | Nessler’s reagent method | / | 252.4 | [31] |
| W_18_O_49_/g-C_3_N_4_ | 300 W Xe lamp  (full spectrum) | / | Nessler’s reagent method | methanol | 64.8 | [32] |
| Fe-W_18_O_49_/NH_2_-MIL-125 | 300 W Xe lamp  (full spectrum) | 200 mW cm^−2^ | Indophenol blue method | Na_2_SO_3_ | 128.2 | [33] |
| Ru-WO_3–_*_x_*/CoO*_x_* | 300 W Xe lamp  (full spectrum) | 100 mW cm^−2^ | Ion chromatography | / | 131.95 | This work |

**Table S8.** Performance of other reported sacrificial agent-free photocatalytic nitrogen fixation systems.

| Photocatalyst | Light source | Light intensity | Quantification method | Oxidative product | NH_3_ yield  (μmol g_cat_^–1^ h^–1^) | Reference |
| --- | --- | --- | --- | --- | --- | --- |
| Ru@MIL-125/MnO*_x_* | 300 W Xe lamp (full-spectrum) | / | Indophenol blue method | Oxygen | 10.43 | [34] |
| Fe-TiO_2_-SiO_2_ | 300 W Xe lamp (full spectrum) | / | Nessler’s reagent method | Oxygen | 32 | [35] |
| Au/(BiO)_2_CO_3_ | 300 W Xe lamp (full-spectrum) | 100 mW cm^−2^ | Indophenol blue method | Oxygen | 38.2 | [36] |
| Porous CuFe | 300 W Xe lamp (full-spectrum) | 250 mW cm^−2^ | Nessler’s reagent method | Oxygen | 342 | [37] |
| Au/r-Ti_3_C_2_ | 300 W Xe lamp (full-spectrum) | / | Nessler’s reagent method | Oxygen | 31.8 | [38] |
| CuCr-LDH | 300 W Xe lamp ( *λ* >400 nm) | / | Nessler’s reagent method | Oxygen | 57.1 | [39] |
| AuRu_0.31_ | 300 W Xe lamp (full-spectrum) | 400 mW cm^−2^ | Ion chromatography | •OH | 101.4 | [40] |
| MIL-125−NH_2_@Co-HHTP | 300 W Xe lamp (full-spectrum) | / | ^1^H NMR | Oxygen | 260 | [41] |
| UiO-66(SH)_2_-200 | 300 W Xe lamp (full-spectrum) | / | Ion chromatography | Oxygen | 32.4 | [42] |
| Graphene-embedded Ce-UiO-66 | 365 nm LED | 3.5 mW cm^−2^ | Ammonia gas sensor | / | 110.24 | [43] |

**Table S9.** ICP-OES analysis results of Ru-WO_3−_*_x_*/CoO*_x_* before and after the cycling experiment.

| Sample | Mass fraction of Ru (wt%) | Mass fraction of Co (wt%) |
| --- | --- | --- |
| Before reaction | 0.37 | 2.14 |
| After reaction | 0.32 | 1.89 |

**Table S10**. Summary of the production rates, nitrogen selectivities, and electron gain/loss conservation analysis for the different catalysts.

| Sample | WO_3−_*_x_* | Ru-WO_3−_*_x_* | WO_3−_*_x_*/CoO*_x_* | Ru-WO_3−_*_x_*/CoO*_x_* |
| --- | --- | --- | --- | --- |
| NH_3_ production rate (μmol g^−1^ h^−1^) | 11.95 | 68.70 | 43.75 | 131.95 |
| O_2_ production rate (μmol g^−1^ h^−1^) | 10.10 | 42.66 | 49.63 | 95.7 |
| H_2_ production rate (μmol g^−1^ h^−1^) | 11.12 | 10.37 | 38.31 | 15.84 |
| H_2_O_2_ production rate (μmol g^−1^ h^−1^) | 3.31 | 13.98 | 11.31 | 12.96 |
| N_2_H_4_ production rate (μmol g^−1^ h^−1^) | 1.11 | 4.57 | 3.31 | 6.88 |
| NO_3_^−^ production rate (μmol g^−1^ h^−1^) | 2.44 | 7.41 | 2.71 | 6.01 |
| NO_2_^−^ production rate (μmol g^−1^ h^−1^) | 0.66 | 2.34 | 0.76 | 1.87 |
| N selectivity | 69.20% | 78.43% | 81.26% | 85.91% |
| Electron consumption rate (μmol g^−1^ h^−1^) | 62.53 | 245.12 | 221.11 | 455.05 |
| Hole consumption rate (μmol g^−1^ h^−1^) | 61.20 | 242.67 | 236.97 | 444.38 |

**4 References**

[1] S. H. Jia, L. B. Zhang, H. L. Liu, et al., “Upgrading of Nitrate to Hydrazine through Cascading Electrocatalytic Ammonia Production with Controllable N-N Coupling,” *Nat. Commun.* **2024**, *15*, 8567, https://doi.org/10.1038/s41467-024-52825-1

[2] H. Y. Bai, S. H. Lam, J. H. Yang, et al., “A Schottky-barrier-free Plasmonic Semiconductor Photocatalyst for Nitrogen Fixation in a “One-stone-two-birds” Manner,” *Adv. Mater.* **2022**, *34*, 2104226, https://doi.org/10.1002/adma.202104226

[3] J. Q. Yan, T. Wang, G. J. Wu, et al., “Tungsten Oxide Single Crystal Nanosheets for Enhanced Multichannel Solar Light Harvesting,” *Adv. Mater.* **2015**, *27*, 1580–1586, https://doi.org/10.1002/adma.201404792

[4] P. Hohenberg, W. Kohn, “Inhomogeneous Electron Gas,” *Phys. Rev.* **1964**, *136*, B864–B871, https://doi.org/10.1103/PhysRev.136.B864

[5] W. Kohn, L. J. Sham, “Quantum Density Oscillations in an Inhomogeneous Electron Gas,” *Phys. Rev.* **1965**, *137*, A1697–A1705, https://doi.org/10.1103/PhysRev.137.A1697

[6] G. Kresse, J. Furthmüller, “Efficient Iterative Schemes for Ab Initio Total-energy Calculations Using a Plane-wave Basis Set,” *Phys. Rev. B* **1996**, *54*, 11169–11186, https://doi.org/10.1103/PhysRevB.54.11169

[7] G. Kresse, D. Joubert, “From Ultrasoft Pseudopotentials to the Projector Augmented-Wave Method,” *Phys. Rev. B* **1999**, *59*, 1758–1775, https://doi.org/10.1103/PhysRevB.59.1758

[8] P. E. Blöchl, “Projector Augmented-Wave Method,” *Phys. Rev. B* **1994**, *50*, 17953–17979, https://doi.org/10.1103/PhysRevB.50.17953

[9] K. An, B. Y. Wu, J. T. Hu, et al., “Schottky‐Barrier‐Free Plasmonic WO_3_‐Based Photocatalysts for Simultaneous N_2_ Fixation and H_2_O_2_ Generation,” *Adv. Mater*. **2025**, e15476, https://doi.org/10.1002/adma.202515476

[10] F. Raziq, A. Aligayev, H. Shen, et al., “Exceptional Photocatalytic Activities of rGO Modified (B,N) Co-Doped WO_3_, Coupled with CdSe QDs for One Photon Z-Scheme System: A Joint Experimental and DFT Study,” *Adv. Sci*. **2022**, *9*, 2102530, https://doi.org/10.1002/advs.202102530

[11] J. P. Perdew, K. Burke, M. Ernzerhof, “Generalized Gradient Approximation Made Simple,” *Phys. Rev. Lett.* **1996**, *77*, 3865–3868, https://doi.org/10.1103/PhysRevLett.77.3865

[12] R. Kishore, X. Cao, X. Q. Zhang, A. B.-Hütter, “Electrochemical Water Oxidation on WO_3_ Surfaces: A Density Functional Theory Study,” *Catal. Today* **2019**, *321*, 94, https://doi.org/10.1016/j.cattod.2018.02.030

[13] Y. Liu, Z. Shen, Z. Yin, et al., “High‐Throughput Theoretical Screening of Single‐Atom Catalysts for Electrochemical Urea Synthesis,” *Angew. Chem. Int. Ed.* **2025**, e16299, https://doi.org/10.1002/anie.202516299

[14] W.-T. Geng, Y.-C. Liu, N. Xu, G. Tang, Y. Kawazoe, V. Wang, “Empowering Materials Science with VASPKIT: A Toolkit for Enhanced Simulation and Analysis,” *Nat. Protoc*. **2025**, *20*, 3143–3169, https://doi.org/10.1038/s41596-025-01160-w

[15] R. Nelson, C. Ertural, J. George, V. L. Deringer, G. Hautier, R. Dronskowski, “LOBSTER: Local Orbital Projections, Atomic Charges, and Chemical-Bonding Analysis from Projector-Augmented-Wave-Based Density-Functional Theory,” *J. Comput. Chem.* **2020**, *41*, 1931–1940, https://doi.org/10.1002/jcc.26353

[16] G. Henkelman, A. Arnaldsson, H. Jónsson, “A Fast and Robust Algorithm for Bader Decomposition of Charge Density,” *Comp. Mater. Sci.* **2006**, *36*, 354–360, https://doi.org/10.1016/j.commatsci.2005.04.010

[17] E. Sanville, S. D. Kenny, R. Smith, G. Henkelman, “Improved Grid-Based Algorithm for Bader Charge Allocation,,” *J. Comput. Chem.* **2007**, *28*, 899–908, https://doi.org/10.1002/jcc.20575

[18] J. D. Chen, C. H. Chen, M. K. Qin, et al., “Reversible Hydrogen Spillover in Ru-WO_3-_*_x_* Enhances Hydrogen Evolution Activity in Neutral pH Water Splitting,” *Nat. Commun.* **2022**, *13*, 5382, https://doi.org/10.1038/s41467-022-33007-3

[19] B. Thomas, B. W. Peng, X. X. Huang, T. Asefa, “Improving the Electrocatalytic Activity of Cobalt Oxide with Bismuth for Acidic Oxygen Evolution Reaction,” *J. Mater. Chem. A* **2024**, *12*, 22528–22538, https://doi.org/10.1039/D4TA02845G

[20] N. Zhang, A. Jalil, D. X. Wu, et al., “Refining Defect States in W_18_O_49_ by Mo Doping: A Strategy for Tuning N_2_ Activation towards Solar-Driven Nitrogen Fixation,” *J. Am. Chem. Soc.* **2018**, *140*, 9434–9443, https://doi.org/10.1021/jacs.8b02076

[21] Y. D. Zhang, T. T. Hou, Q. Xu, et al., “Dual-Metal Sites Boosting Polarization of Nitrogen Molecules for Efficient Nitrogen Photofixation,” *Adv. Sci.* **2021**, *8*, 2100302, https://doi.org/10.1002/advs.202100302

[22] B. Hu, B.-H. Wang, L. Chen, et al., “Electronic Modulation of the Interaction between Fe Single Atoms and WO_2.72–_*_x_* for Photocatalytic N_2_ Reduction,” *ACS Catal.* **2022**, *12*, 11860–11869, https://doi.org/10.1021/acscatal.2c03367

[23] G. J. Dong, X. J. Huang, Y. P. Bi, “Anchoring Black Phosphorus Quantum Dots on Fe-Doped W_18_O_49_ Nanowires for Efficient Photocatalytic Nitrogen Fixation,” *Angew. Chem. Int. Ed.* **2022**, *61*, e202204271, https://doi.org/10.1002/anie.202204271

[24] P. Qiu, C. Huang, G. Dong, et al., “Plasmonic Gold Nanocrystals Simulated Efficient Photocatalytic Nitrogen Fixation over Mo Doped W_18_O_49_ Nanowires,” *J. Mater. Chem. A* **2021**, *9*, 14459–14465, https://doi.org/10.1039/D1TA03339E

[25] P. F. Xia, X. C. Pan, S. L. Jiang, et al., “Designing a Redox Heterojunction for Photocatalytic “Overall Nitrogen Fixation” under Mild Conditions,” *Adv. Mater.* **2022**, *34*, 2200563, https://doi.org/10.1002/adma.202200563

[26] L. J. Xiong, Y. J. Hu, Y. Yang, et al., “Electron Pump Strengthened Facet Engineering: Organic Half-Metallic C(CN)_3_ Enclosed (100) Facet Exposed WO_3_ for Efficient and Selective Photocatalytic Nitrogen Fixation,” *Appl. Catal. B Environ.* **2022**, *317*, 121660, https://doi.org/10.1016/j.apcatb.2022.121660

[27] L. B. Wang, S. Y. Wang, D. H. Cui, et al., “Z-Scheme Heterojunctions with Double Vacancies Semiconductors Moo_3−_*_x_* and Fe-Doped W_18_O_49_ For Photocatalytic Nitrogen Fixation,” *J. Alloys Compd.* **2022**, *927*, 167003, https://doi.org/10.1016/j.jallcom.2022.167003

[28] H. Shang, Y. J. Wang, H. B. Jia, et al., “Constructing Asymmetric Active Sites on Defective Ru/W_18_O_49_ for Photocatalytic Nitrogen Fixation,” *Catal. Sci. Technol.* **2023**, *13*, 854–861, https://doi.org/10.1039/D2CY01859D

[29] Z. X. Yang, J. Q. Wang, J. T. Wang, et al., “2D WO_3–_*_x_* Nanosheet with Rich Oxygen Vacancies for Efficient Visible-Light-Driven Photocatalytic Nitrogen Fixation,” *Langmuir* **2022**, *38*, 1178–1187, https://doi.org/10.1021/acs.langmuir.1c02862

[30] X. C. Hui, L. F. Li, Q. N. Xia, et al., “Interface Engineered Sb_2_O_3_/W_18_O_49_ Heterostructure for Enhanced Visible-Light-Driven Photocatalytic N_2_ Reduction,” *Chem. Eng. J.* **2022**, *438*, 135485, https://doi.org/10.1016/j.cej.2022.135485

[31] D. H. Cui, S. Y. Wang, X. Yang, L. Xu, F. Y. Li, “Fabrication of Ultrafine Cu_2_O Nanoparticles on W_18_O_49_ Ultra-Thin Nanowires by In-Situ Reduction for Highly Efficient Photocatalytic Nitrogen Fixation,” *Small* **2024**, *20*, 2306229, https://doi.org/10.1002/smll.202306229

[32] C. H. Huang, Y. Ma, Q. Cheng, et al., “Effective 1D/2D Nanostructured S-Scheme W_18_O_49_/G-C_3_N_4_ Heterojunction Photocatalyst Fabrication for Improved Photocatalytic Nitrogen Fixation Performance,” *Appl. Surf. Sci.* **2024**, *659*, 159952, https://doi.org/10.1016/j.apsusc.2024.159952

[33] Z. H. Hou, L. B. Wang, G. Liu, et al., “Construct Electron Transport Path through NH_2_-MIL-125 Modified Fe-Doped W_18_O_49_ Nanowires to Enhance Photocatalytic Nitrogen Fixation Performance,” *J. Alloys Compd.* **2024**, *973*, 172940, https://doi.org/10.1016/j.jallcom.2023.172940

[34] C. J. Wang, S. Wang, Y. Ping, et al., “Ru@MIL-125/MnO*_x_* Metal-Organic-Framework-Based Cocatalysts for Photocatalytic Nitrogen Fixation,” *Appl. Catal. B Environ.* **2024**, *347*, 123781, https://doi.org/10.1016/j.apcatb.2024.123781
[35] S. Q. Wu, Z. Y. Chen, W. H. Yue, et al., “Single-Atom High-Valent Fe(IV) for Promoted Photocatalytic Nitrogen Hydrogenation on Porous TiO_2_-SiO_2_,” *ACS Catal.* **2021**, *11*, 4362–4371, https://doi.org/10.1021/acscatal.1c00072
[36] C. L. Xiao, H. Hu, X. Y. Zhang, D. R. MacFarlane, “Nanostructured Gold/Bismutite Hybrid Heterocatalysts for Plasmon-Enhanced Photosynthesis of Ammonia,” *ACS Sustainable Chem. Eng.* **2017**, *5*, 10858–10863, https://doi.org/10.1021/acssuschemeng.7b02788
[37] T. T. Hou, L. L. Chen, Y. Xin, et al., “Porous CuFe for Plasmon-Assisted N_2_ Photofixation,” *ACS Energy Lett.* **2020**, *5*, 2444–2451, https://doi.org/10.1021/acsenergylett.0c00959
[38] B. B. Chang, Y. Z. Guo, D. Wu, L. Li, B. C. Yang, J. F. Wang, “Plasmon-Enabled N_2_ Photofixation on Partially Reduced Ti_3_C_2_ MXene,” *Chem. Sci.* **2021**, *12*, 11213–11224, https://doi.org/10.1039/D1SC02772G
[39] Y. F. Zhao, Y. X. Zhao, G. I. N. Waterhouse, et al., “Layered-Double-Hydroxide Nanosheets as Efficient Visible-Light-Driven Photocatalysts for Dinitrogen Fixation,” *Adv. Mater.* **2017**, *29*, 1703828, https://doi.org/10.1002/adma.201703828
[40] C. Y. Hu, X. Chen, J. B. Jin, et al., “Surface Plasmon Enabling Nitrogen Fixation in Pure Water through a Dissociative Mechanism under Mild Conditions,” *J. Am. Chem. Soc.* **2019**, *141*, 7807–7814, https://doi.org/10.1021/jacs.9b01375

[41] L. Yuan, C. Tang, P. Y. Du, et al., “Nanoporous Heterojunction Photocatalysts with Engineered Interfacial Sites for Efficient Photocatalytic Nitrogen Fixation,” *Angew. Chem. Int. Ed.* **2024**, *63*, e202412340, https://doi.org/10.1002/anie.202412340

[42] B. B. Guo, X. Y. Cheng, Y. Tang, et al., “Dehydrated UiO-66(SH)_2_: The Zr−O Cluster and Its Photocatalytic Role Mimicking the Biological Nitrogen Fixation,” *Angew. Chem. Int. Ed.* **2022**, *61*, e202117244, https://doi.org/10.1002/anie.202117244

[43] S. X. Liu, Z. Y. Teng, H. Liu, et al., “A Ce-UiO-66 Metal–Organic Framework-Based Graphene-Embedded Photocatalyst with Controllable Activation for Solar Ammonia Fertilizer Production,” *Angew. Chem. Int. Ed.* **2022**, *61*, e202207026, https://doi.org/10.1002/anie.202207026
